# Chaperonin TRiC/CCT supports mitotic exit and entry into endocycle in *Drosophila*

**DOI:** 10.1371/journal.pgen.1008121

**Published:** 2019-04-29

**Authors:** Yuya Ohhara, Aki Nakamura, Yuki Kato, Kimiko Yamakawa-Kobayashi

**Affiliations:** 1 School of Food and Nutritional Sciences, University of Shizuoka, Shizuoka, Japan; 2 Graduate School of Integrated Pharmaceutical and Nutritional Sciences, University of Shizuoka, Shizuoka, Japan; Katholieke Universiteit Leuven, BELGIUM

## Abstract

Endocycle is a commonly observed cell cycle variant through which cells undergo repeated rounds of genome DNA replication without mitosis. Endocycling cells arise from mitotic cells through a switch of the cell cycle mode, called the mitotic-to-endocycle switch (MES), to initiate cell growth and terminal differentiation. However, the underlying regulatory mechanisms of MES remain unclear. Here we used the *Drosophil*a steroidogenic organ, called the prothoracic gland (PG), to study regulatory mechanisms of MES, which is critical for the PG to upregulate biosynthesis of the steroid hormone ecdysone. We demonstrate that PG cells undergo MES through downregulation of mitotic cyclins, which is mediated by Fizzy-related (Fzr). Moreover, we performed a RNAi screen to further elucidate the regulatory mechanisms of MES, and identified the evolutionarily conserved chaperonin TCP-1 ring complex (TRiC) as a novel regulator of MES. Knockdown of TRiC subunits in the PG caused a prolonged mitotic period, probably due to impaired nuclear translocation of Fzr, which also caused loss of ecdysteroidogenic activity. These results indicate that TRiC supports proper MES and endocycle progression by regulating Fzr folding. We propose that TRiC-mediated protein quality control is a conserved mechanism supporting MES and endocycling, as well as subsequent terminal differentiation.

## Author summary

Endocycle, a cell cycle variant consisting of DNA replication and Gap phases without a mitotic phase, is widespread in nature. Endocycling cells arise from proliferating cells through a switch of the cell cycle mode called mitotic-to-endocycle switching (MES). While the molecular mechanisms regulating progression of the mitotic cell cycle and endocycle have been well studied, the regulatory mechanism of MES and its impact on cell differentiation processes remains poorly understood. Here we used the *Drosophil*a steroidogenic organ to uncover the regulatory factors of MES, and our genetic analyses identified the evolutionarily conserved chaperonin TRiC as a novel regulator of MES and subsequent steroidogenesis. Given that TRiC supports proper folding of numerous proteins including cell cycle regulators, TRiC-mediated protein quality control will be a fundamental mechanism supporting a switch of the cell cycle mode that promotes terminal differentiation.

## Introduction

A tightly controlled cell cycle is a fundamental system for survival in every organism. The best-known cell cycle mode is mitotic cell cycle, which is achieved through a sequence of distinct phases including genome DNA synthesis (S), mitotic (M), and intervening Gap (G) phases. Moreover, endocycle, a cell cycle variant without M phase, is commonly observed in protozoa, plants, and animals [1, 2]. Endocycling cells undergo repeated rounds of S and G phases without a M phase, which gives rise to polyploidy of genome DNA. Endocycle and polyploidy are closely associated with cell growth; in some cell types, progression of endocycling is required for their terminal differentiation [2–4]. Moreover, polyploid genomic DNA has been observed in approximately 37% of all human tumors [5], and several lines of evidence point to the importance of endocycle in tumor development and survival [6–8]. Thus, elucidation of the underlying mechanisms regulating initiation and progression of endocycle is a key step to understanding the role of endocycle in normal and pathological cellular processes.

The important question in endocycle regulation is how the transition from cell division to endocycle is achieved. Endocycling cells arise from diploid cells through a switch of the cell cycle mode, called the mitotic-to-endocycle switch (MES) [1]. At the molecular level, MES is accomplished by downregulation of mitotic cyclin-dependent kinases (M-Cdks), which leads to the M phase being bypassed. M-Cdk is suppressed through degradation of its binding partners, called mitotic cyclins, including Cyclin A (CycA) and B (CycB). Mitotic cyclins are recognized by Fizzy-related [Fzr, a.k.a. CDH1 (CDC20 Homologue 1)], an activator of a multi-subunit ubiquitin ligase anaphase promoting complex/cyclosome (APC/C), to be polyubiquitinated, and then degraded through the ubiquitin-proteasome pathway [1, 9]. Fzr triggers MES to support the progression of numerous biological events including morphogenesis, growth, and tissue repair in insects, plants, and mammals [1, 10–18]. Fzr-mediated degradation of mitotic cyclins is commonly observed during MES [1, 11, 16, 18], whereas identified upstream regulators of Fzr are diverse among species [1]. It therefore remains unclear whether there are evolutionary conserved regulatory mechanisms of Fzr expression. In addition, it is largely unknown how mitotic exit and endocycle progression are cooperatively regulated.

In this study, we focused on the *Drosophila* prothoracic gland (PG), an endocrine organ composed of polyploid endocycling cells, for dissecting the molecular mechanisms supporting MES and the progression of endocycle. The PG produces ecdysone, the primary insect steroid hormone that triggers initiation and progression of metamorphosis [19, 20]. In *Drosophila* and other higher Diptera, the PG is part of the ring gland (RG), an endocrine organ complex that comprises the PG, corpus cardiacum (CC), and corpus allatum (CA) [19]. The PG expresses a set of ecdysone biosynthetic genes including Neverland (Nvd), Spookier (Spok), Shroud (Sro), Phantom (Phm), Disembodied (Dib), and Shadow (Sad), whose expression is upregulated before the initiation of metamorphosis to enhance the ecdysteroidogenic activity [21–23]. Ecdysone secreted from the PG is converted into its active form, 20-hydroxyecdysone (20E), in peripheral tissues [19]. In *Drosophila*, PG cells undergo repeated rounds of endocycling during the larval stage, and endocycle progression is essential for activating ecdysone biosynthesis in the PG [22]. After at least three rounds of endocycle (when the C value reaches 32), biosynthesis of ecdysone is initiated to induce the larval-to-pupal metamorphic transition [22]. When endocycle progression is impaired in the PG, ecdysone biosynthesis is not upregulated, and the larva cannot transit into the pupal stage [22]. Because this ‘larval arrest’ phenotype is readily recognized, PG-selective genetic analysis can be a potentially effective approach to screen novel regulators of MES and endocycle progression. Its relatively large cell size and low cell number (its width is about 200 μm at the late larval stage and the cell number is around 50) also make the PG an attractive model for investigating MES and endocycle progression.

Here we show that PG cells undergo MES through downregulation of mitotic cyclins mediated by Fzr. Fzr protein expression is increased during the MES period in the PG, and knockdown of *fzr* in the PG causes a block of MES and subsequent ecdysone biosynthesis owing to upregulation of mitotic cyclins. Furthermore, we performed a PG-selective RNAi screen to further elucidate the regulatory mechanism of MES, and identified TCP-1 ring complex (TRiC), a molecular chaperonin complex, as a novel MES regulator. TRiC-deficient PG cells showed a prolonged mitotic period, probably due to impaired nuclear translocation of Fzr, which also caused loss of ecdysteroidogenic activity. Our genetic study provides an important basis for understanding the regulatory mechanisms of endocycle initiation and progression.

## Result

### PG cells undergo MES

**Fig 1:**
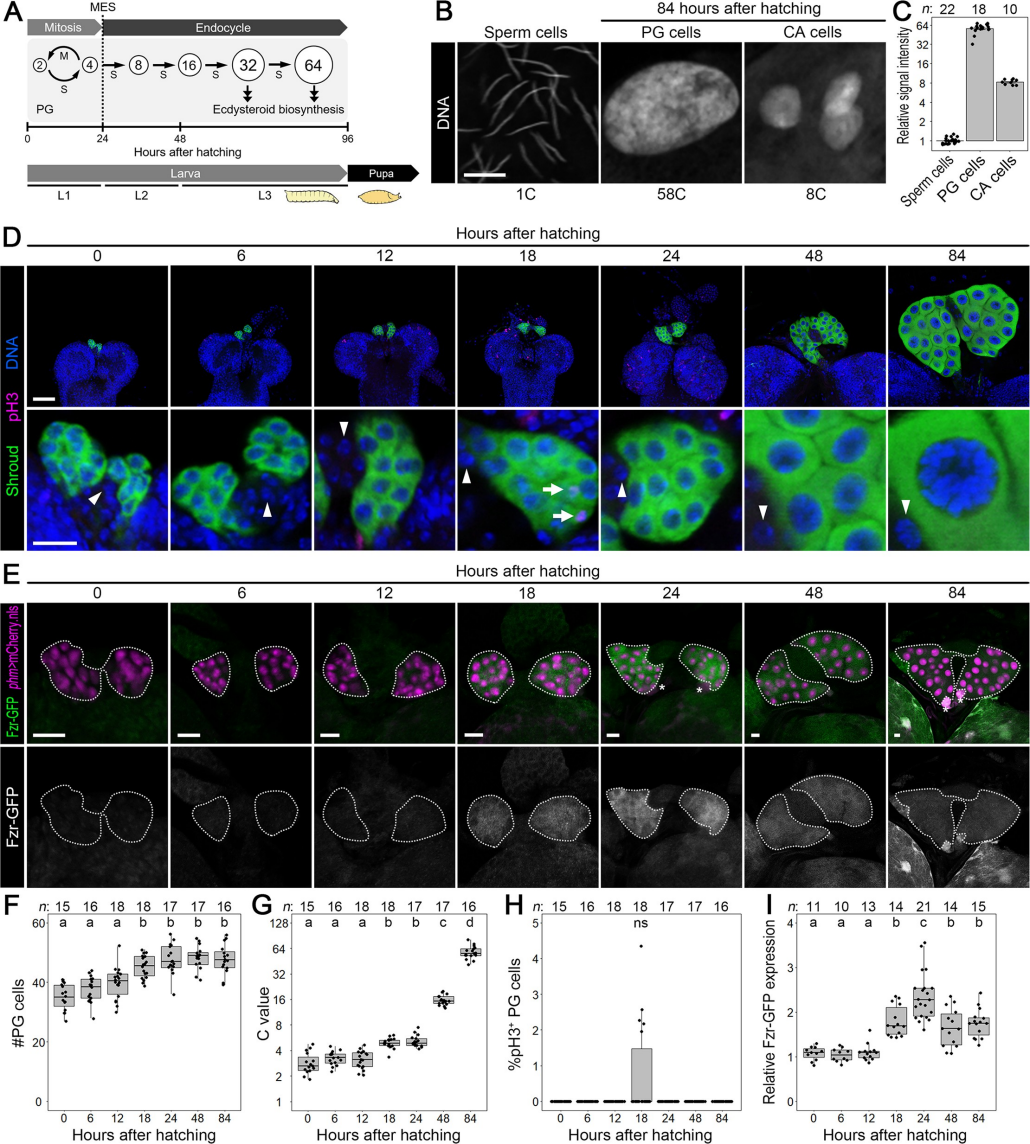
PG cells undergo MES. **(A)** Schematic diagram of the cell cycle mode in the PG during larval development. **(B)** Adult sperm cells (left panel), larval PG (middle panel) and CA cells (right panel) at 84 hAH of wild-type strain *Oregon R*. DNA was stained by Hoechst. The mean C value is shown below each panel (see Fig 1C). Scale bars: 10 μm. **(C)** Scatter plots with a mean value of relative signal intensity of Hoechst in *Oregon R* sperm, PG, and CA cells. The mean C value in sperm cells was normalized to 1, and accordingly, the mean C values in PG and CA cells at 84 hAH were set to 58 and 8, respectively. Sample sizes (the number of sperm, PG, and CA cells) are shown above each column. **(D)** The PG (upper panels) and PG cells in higher magnification (lower panels) of *Oregon R* at indicated time points. PG cells, mitotic pH3-positive cells, and DNA were detected by anti-Sro antibody (green), anti-pH3 antibody (magenta), and Hoechst (blue), respectively. The arrows and arrowheads indicate pH3-positive PG cells and the nuclei of CA cells, respectively. Scale bars: 50 μm (upper panels) and 10 μm (lower panels). **(E)** Fzr-GFP expression in the PG at indicated time points. Fzr-GFP (green and white in the upper and lower panels, respectively) was detected using anti-GFP antibody at indicated stages. PG cells are labeled by mCherry.nls driven by PG-selective *phm-Gal4* (magenta in the upper panels). The PGs are indicated by dotted lines. The asterisks indicate CC cells expressing DsRed driven by *eyeless* promoter: 3xP3-dsRed marker in Fzr-GFP transgene. Scale bars: 10 μm. **(F–H)** Scatter and box plots showing the cell number (F), the C value (G), and the percentage of pH3-positive cells (H) in the PG of *Oregon R* at indicated time points. Different lowercase letters indicate statistically significant differences (*P* < 0.05; Steel–Dwass test; see S1 Table). ns, not significant (*P* > 0.05). Box plots indicate the median (bold line), the 25th and 75th percentiles (box edges), and the range (whiskers). Dot plots show all data points individually. The mean C value at 84 hAH was normalized to 58 (see Fig 1C), and all data points were normalized accordingly. Sample sizes (the number of PGs) are shown above each column. **(I)** Scatter and box plots showing the relative expression level of Fzr-GFP in the PG at indicated stages. Different lowercase letters indicate statistically significant differences (*P* < 0.05; Steel–Dwass test; see S1 Table). Box and dot plots as in Fig 1C–1E. Sample sizes (the number of PGs) are shown above each column.

In wild type strain *Oregon R*, the PG increases its cell number during the 1st instar larval stage [L1, from 0 to 24 hours after hatching (hAH)] [22]. During the 2nd (L2, from 24 to 48 hAH) and the 3rd (L3, from 48 to 96 hAH) instar larval stages, in contrast, the PG cell number does not increase while the DNA content continues to increase and the C value reaches 32–64C by the late L3 stage, 84–96 hAH (Fig 1A) [22]. This suggests that PG cells undergo cell division during L1, execute MES at around 24 hAH, and undergo 3–4 rounds of endocycle during the L2 and L3 stages (Fig 1A). First we determined that the mean C value in the PG of *Oregon R* at 84 hAH was 58 (Fig 1B and 1C). In addition, the C values in two transgenic control lines at 96 hAH were 53 and 54, respectively (S1A and S1B Fig). These results indicate that PG cells undergo approximately four rounds of endocycling. Considering that these C values are less than 64, which is achieved by four complete rounds of endocycling, it is suggested that replication of genomic DNA in PG cells is incomplete, which is known as under-replication [1]. We next tested whether DNA replication was suppressed in the heterochromatic region, in which under-replication is commonly observed [1]. 5-Bromodeoxyuridine (BrdU), an analog of thymidine used as a marker of replication activity, was incorporated into the DNA dense-core heterochromatic region of PG cells at 84 hAH, but its frequency was less than the early and middle S-phases (S1C and S1D Fig). This suggests that the genomic DNA of PG cells exhibits under-replication but the replication in the under-replicated region is not severely impaired.

Next, we investigated the cell number, the DNA content, and expression of the mitotic marker pH3 (histone H3 phosphorylated at serine 10) in the PG of *Oregon R* during larval development. The PG cell number was increased during 12 to 18 hAH (Fig 1D and 1F), suggesting that mitotic cell cycle in the PG is active approximately during 12 to 18 hAH. In contrast, no significant increase in the PG cell number was observed at 24 hAH and thereafter (Fig 1D and 1F), and the C value started to increase at 18 hAH (Fig 1D and 1G). These results suggest that some, but not all, PG cells start MES from 18 hAH. In contrast, pH3-positive PG cells were detectable at 18 hAH (Fig 1D and 1H), but we could not detect a statistically significant increase in the percentage of pH3-positive PG cells at 18 hAH (Fig 1H). We also failed to detect pH3 expression in the PG at 12 hAH (Fig 1D and 1H), probably reflecting transient pH3 expression owing to rapid mitotic cycles. To clarify cell cycle phase in PG cells during 0 to 24 hAH, we used Fly Fluorescent Ubiquitin-based Cell Cycle Indicator (Fly-FUCCI) driven by PG-selective *phantom-22-Gal4* (*phm-Gal4*) [24, 25]. The Fly-FUCCI components GFP-fused E2F1^1-230^ (GFP.E2F1) and mRFP1-fused CycB^1-266^ (mRFP1.CycB) are expressed in patterns consistent with the presence of G1, S, and G2/M cells [24] (S1E and S1F Fig). The percentage of mRFP.CycB-positive/GFP.E2F1-negative cells (= S phase) reached the maximum level at 6 hAH (S1G and S1H Fig), indicating that S-phase of mitotic cell cycle is active at around 6 hAH. In contrast, the percentage of both mRFP.CycB and GFP.E2F1-positive cells (= G2/M phases) was increased at 12 and 18 hAH (S1G and S1H Fig), confirming that mitosis is active between 12 and 18 hAH. Actually, a small number of mRFP.CycB and GFP.E2F1-positive cells showed a metaphase-like nuclear shape at 12 and 18 hAH (arrows in S1G Fig). Furthermore, mRFP.CycB-positive PG cells were dramatically reduced at 24 hAH, and the majority of PG cells was mRFP.CycB-negative/GFP.E2F1-positive at 24 hAH (S1G and S1H Fig), indicating that mitotic cell cycle is downregulated by 24 hAH. Taken together, these observations indicate that mitotic cell cycle is active during a narrow time window, approximately between 12 and 18 hAH, and that PG cells execute MES by 24 hAH. Moreover, Fzr expression, which was visualized by GFP-fused Fzr (Fzr-GFP), was upregulated at 18 hAH and reached the maximum level at 24 hAH (Fig 1E and 1I), supporting the idea that Fzr-mediated MES in the PG starts at around 18 hAH and that PG cells execute MES by 24 hAH. In addition, Fzr-GFP was detectable at lower levels during later stages (Fig 1E and 1I), suggesting that Fzr blocks mitosis continuously during endocycling in the PG.

In contrast to the PG, pH3 expression was not observed in the CA (Fig 1D; CA cells are indicated by the arrowheads), another endocrine organ in the RG complex. This suggests that CA cells do not undergo cell division during the larval stage. However, the mean C value in CA cells reached around 8 at 84 hAH (Fig 1B and 1C), indicating that CA cells undergo a round of endocycle. Actually the DNA content in CA cells seems to be increased during 24 to 84 hAH (arrowheads in Fig 1D), suggesting that CA cells undergo endocycle during the L2 and L3 stages. To test this possibility, expression of S-phase activator Cyclin E (CycE) and incorporation of BrdU were observed in CA cells labeled by red fluorescent protein (RFP) expressed under the control of *jhamt-Gal4*. CycE-positive CA cells were detected at 48, 72, and 96 hAH (S2A and S2B Fig), and BrdU was incorporated into CA cells in these stages (S2C and S2D Fig), indicating that CA cells undergo endocycle during the L2/L3 stages. Because the percentage of BrdU-positive CA cells was reduced at 96 hAH (S2C and S2D Fig), CA cells are likely to terminate endocycle by the end of the larval stage.

### Fzr-mediated downregulation of mitotic cyclins is required for MES and ecdysone biosynthesis in the PG

**Fig 2:**
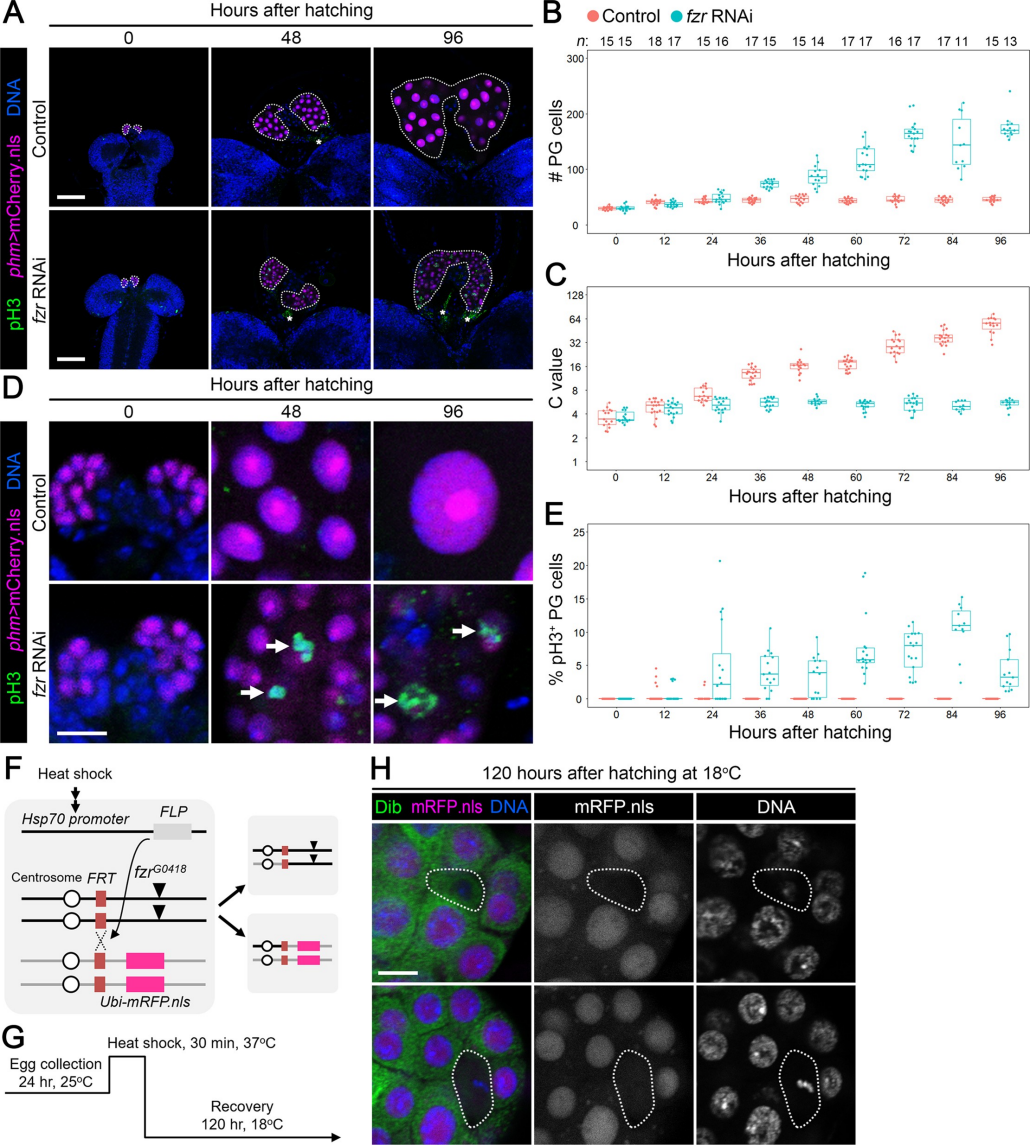
Fzr is required for MES in the PG. **(A)** The PG of the controls (*phm>dicer2 mCherry*.*nls*) (upper panels) and *fzr* RNAi (*phm>dicer2 mCherry*.*nls fzr-RNAi*) (lower panels) at indicated time points. pH3 and DNA were detected by anti-pH3 antibody (green) and Hoechst (blue), respectively, and the nuclei of PG cells were labelled by mCherry.nls (magenta). The PGs are indicated by dotted lines. The asterisks indicate nonspecific anti-pH3 antibody signal around CC cells. Scale bars: 50 μm. **(B and C)** Scatter and box plots showing the cell number (B) and the C value (C) in the PG of the controls (red) and *fzr* RNAi animals (blue) at indicated stages. *P*-values for all pairwise comparisons are shown in S1 Table (Steel–Dwass test). Box and dot plots as in Fig 1C–1E. The mean C value of control at 96 hAH was set to 54 (see S1B Fig). Sample sizes (the number of PGs) are shown above each column. **(D)** PG cells in higher magnification of the controls (upper panels) and *fzr* RNAi animals (lower panels) at indicated stages. The arrows indicate pH3-positive PG cells. Scale bars: 10 μm. **(E)** Scatter and box plots showing the percentage of pH3-positive PG cells of the controls (red) and *fzr* RNAi animals (blue) at indicated stages. *P*-values for all pairwise comparisons are shown in S1 Table (Steel–Dwass test). Sample sizes are the same as in B and C. **(F)** Schematic diagram of FLP/FRT system. Heat-shock-induced FLP recombinase recognizes *FRT* sites to induce trans-chromosome recombination between chromosome arms carrying *fzr*^*G0418*^ and mCherry.nls, which leads to wild-type and homozygous mutant progenies. **(G)** Diagram of temperature-shift experiment. Newly-hatched larvae are heat-shocked at 37°C for 30 min, and then recovered at 18°C until 120 hAH. **(H)** *fzr*^*G0418*^/*fzr*^*G0418*^ clone PG cells. *fzr*^*G0418*^/*fzr*^*G0418*^ homozygous mutant cells (indicated by dotted lines) do not express mRFP.nls (magenta and white in the left and middle panels, respectively). The PG and nuclear DNA were labelled by anti-Dib antibody (green in the left panels) and Hoechst (blue and white in the left and right panels, respectively), respectively. Scale bars: 10 μm.

To investigate whether Fzr regulates MES in the PG, PG-selective *phm-Gal4* was used to overexpress RNAi construct against *fzr* in the PG. The nuclei of PG cells were labeled by mCherry carrying the nuclear localization signal (mCherry.nls), and RNAi efficiency was enhanced by overexpression of *dicer2*. In accordance with the result of ploidy measurement, the mean C value of the controls (*phm>dicer2 mCherry*.*nls*) was set to 54 (see S1A and S1B Fig). In the control animals, the PG cell number was slightly increased only during 0 to 12 hAH (Fig 2A and 2B), whereas the C value in the PG started to increase from 24 hAH and continuously increased until 96 hAH (Fig 2A and 2C). In contrast, the PG of *fzr* RNAi animals (*phm>dicer2 mCherry*.*nls fzr-RNAi*) continuously increased its cell number while its C value was around 4C during larval development (Fig 2A–2C). Consistent with these observations, pH3 was continuously detected in the PG of the *fzr* RNAi animals throughout larval development (Fig 2D and 2E). In addition, we confirmed efficient knockdown of Fzr, visualized by Fzr-GFP, along with upregulation of pH3 in the PG of *fzr* RNAi at 24, 48, 72, and 96 hAH (S3 Fig). Furthermore, hypomorphic *fzr*^*G0418*^ mutant clone PG cells, introduced by FLP/FRT recombination induced by heat-shock just after hatching (Fig 2F and 2G), showed a reduced DNA level and, in some mutant clones, metaphase-like DNA distribution (Fig 2H). Taken together, these results indicate that *fzr* is required for MES in the PG. Considering that mitotic cell cycle was not arrested in *fzr* RNAi, we suggest that *fzr* is unnecessary for progression of mitotic cell cycle, which has been described in a previous study [11].

We also confirmed that, as shown in a previous study [22], knockdown of *fzr* in the PG caused L3 arrest (S4A–S4C Fig), reduction in ecdysteroidogenic gene expression (S4D Fig), and a low ecdysteroid level (S4E Fig). Furthermore, 20E administration to *fzr* RNAi animals rescued their defects in pupariation (S4F and S4G Fig). These results indicate that *fzr*-mediated MES in the PG is required for activation of ecdysone biosynthesis and pupariation. However, *fzr* RNAi larvae did not show any defects in the L1-L2 and L2-L3 molting (S4A and S4B Fig), which are also triggered by ecdysone. This confirms that Fzr-mediated MES in the PG is required for pupariation but not for the L1-L2 and L2-L3 molting.

**Fig 3:**
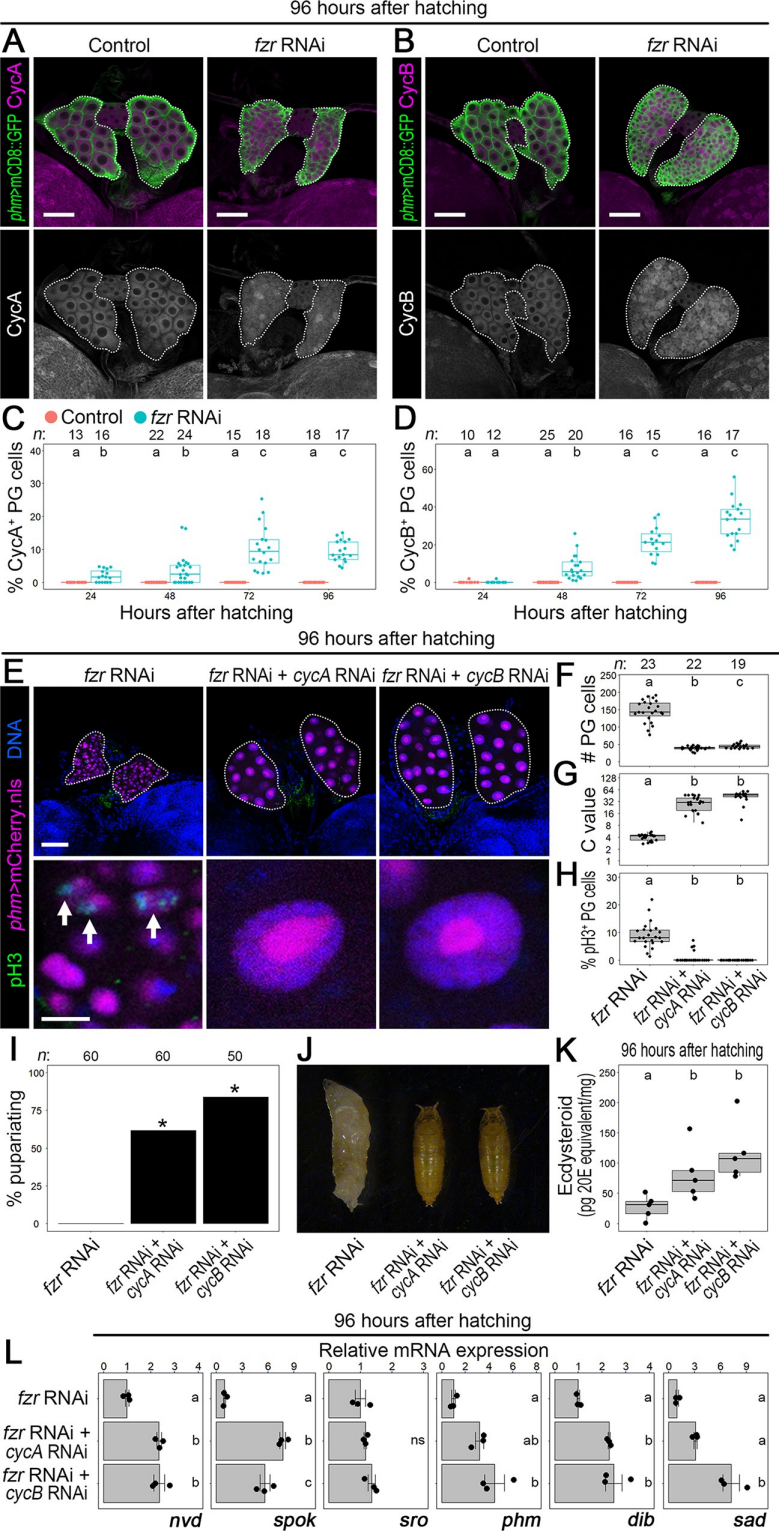
Fzr-mediated downregulation of mitotic cyclins is required for MES and ecdysteroidogenesis in the PG. **(A and B)**. CycA (A) and B expression (B) in the PG of the controls (*phm>dicer2 mCD8*::*GFP*) and *fzr* RNAi larvae (*phm>dicer2 mCD8*::*GFP fzr-RNAi*) at 96 hAH. PG cells were labelled by mCD8::GFP (green in the upper panels) and CycA and B were detected by specific antibodies against each cyclin (magenta and white in the upper and lower panels, respectively). The PGs are indicated by dotted lines. Scale bars = 50 μm. **(C and D)** Scatter and box plots showing the percentage of CycA (C) and CycB (D)-positive PG cells of the controls (red) and *fzr* RNAi animals (blue) at indicated stages. Different lowercase letters indicate statistically significant differences (*P* < 0.05; Steel–Dwass test; see S1 Table). Box and dot plots as in Fig 1C–1E. Sample sizes (the number of PGs) are shown above each column. **(E)** The PG (upper panels) and PG cells in higher magnification (lower panels) of *fzr* RNAi (*phm>dicer2 mCherry*.*nls fzr-RNAi*), *fzr* RNAi + *cycA* RNAi (*phm>dicer2 mCherry*.*nls fzr-RNAi cycA-RNAi*), and *fzr* RNAi + *cycB* RNAi (*phm>dicer2 mCherry*.*nls fzr-RNAi cycB-RNAi*) at 96 hAH. pH3 and DNA were detected by anti-pH3 antibody (green) and Hoechst (blue), respectively, and the nuclei of PG cells were labelled by mCherry.nls (magenta). The PGs are indicated by dotted lines. The arrows indicate pH3-labelled mitotic PG cells. Scale bars: 50 μm (upper panels) and 10 μm (lower panels). **(F–H)** Scatter and box plots showing the cell number (F), the C value (G), and the percentage of pH3-positive cells (H) in the PG of *fzr* RNAi, *fzr* RNAi + *cycA* RNAi, and *fzr* RNAi + *cycB* RNAi animals at 96 hAH. Different lowercase letters indicate statistically significant differences (*P* < 0.05; Steel–Dwass test; see S1 Table). The mean C value of *fzr* RNAi at 96 hAH was set to 4 according to its mean C value at the same time point (Fig 2C). Sample sizes (the number of PGs) are shown above each column.**(I)** Percentages of pupariated *fzr* RNAi (*phm>dicer2 fzr-RNAi*), *fzr* RNAi + *cycA* RNAi (*phm>dicer2 fzr-RNAi cycA-RNAi*), and *fzr* RNAi + *cycB* RNAi animals (*phm>dicer2 fzr-RNAi cycB-RNAi*) are shown. The asterisks indicate statistically significant differences between *fzr* RNAi and *fzr* RNAi + *cycA* RNAi or *fzr* RNAi + *cycB* RNAi (*P* < 0.05; Fisher’s test). Sample sizes (the number of animals) are shown above each column. **(J)** *fzr* RNAi animals arrested at the L3 stage and pupariated *fzr* RNAi + *cycA* RNAi and *fzr* RNAi + *cycB* RNAi animals at 120 hAH. **(K)** Whole-body ecdysteroid levels in *fzr* RNAi, *fzr* RNAi + *cycA* RNAi, and *fzr* RNAi + *cycB* RNAi animals at 96 hAH were measured using ELISA. Scatter and box plots show ecdysteroid level of five independent data sets (10 larvae were pooled in each datum). Different lowercase letters indicate statistically significant differences (*P* < 0.05; Steel–Dwass test; see S1 Table). **(L)** The expression level of ecdysone biosynthetic genes at 96 hAH was measured using qPCR. Scatter plots with average values of triplicate data sets are shown with SE (ten to fifteen larvae were pooled in each datum). Different lowercase letters indicate statistically significant differences (*P* < 0.05; Tukey’s multiple comparison test; see S1 Table).

Next, we investigated whether Fzr triggers MES through downregulation of mitotic cyclins in the PG. In the controls (*phm>dicer2 mCD8*::*GFP*), neither CycA nor B expression was observed in the PG at post-MES stages, including 24, 48, 72, and 96 hAH (Fig 3A–3D, S5 Fig), whereas CycA was upregulated in the PG of *fzr* RNAi animals (*phm>dicer2 mCD8*::*GFP fzr-RNAi*) at the same stages (Fig 3A–3D, S5 Fig). CycB was also detectable in the PG of *fzr* RNAi at 48, 72, and 96 hAH (Fig 3A–3D, S5 Fig). These results suggest that *fzr*-deficient PG cells cannot undergo MES, and ecdysone biosynthesis is inhibited owing to highly expressed mitotic cyclins. To test this possibility, we examined whether knockdown of *cycA* and *B* rescues impaired MES and ecdysteroidogenesis in *fzr* RNAi animals. In contrast to *fzr* RNAi animals (*phm>dicer2 mCherry*.*nls fzr RNAi*) showing a cell number increase and reduced DNA content in the PG (Fig 3E–3G), the C value reached 32–64 and the cell number was restored to around 50 at 96 hAH in the PG of both *fzr* RNAi + *cycA* RNAi (*phm>dicer2 mCherry*.*nls fzr-RNAi cycA-RNAi*) and *fzr* RNAi + *cycB* RNAi animals (*phm>dicer2 mCherry*.*nls fzr-RNAi cycB-RNAi*) (Fig 3E–3G). Consistent with this, the pH3-positive PG cell number was reduced in both *fzr* RNAi + *cycA* RNAi and *fzr* RNAi + *cycB* RNAi animals (Fig 3H). These observations indicate that *fzr* induces MES through inactivation of mitotic cyclins in the PG. Moreover, knockdown of *cycA* or *B* in the PG of *fzr* RNAi animals rescued the developmental arrest at L3 (Fig 3I and 3J) and restored the expression level of ecdysone biosynthetic genes and the ecdysteroid concentration (Fig 3K and 3L). Taken together, these results indicate that the Fzr-mediated reduction of the mitotic cyclin protein level induces MES and subsequent ecdysone biosynthesis in the PG.

### PG-selective RNAi screen to identify novel MES regulators

**Fig 4:**
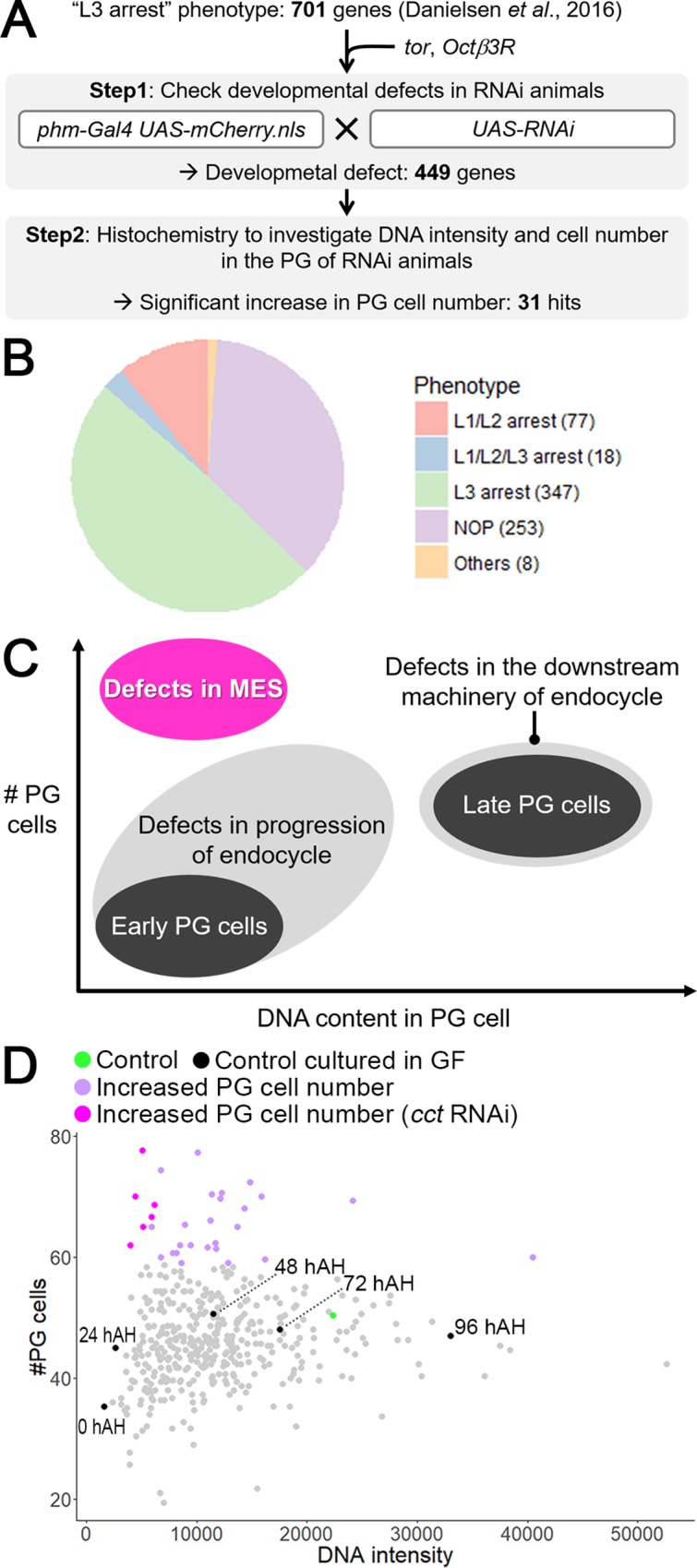
PG-selective RNAi screen to identify novel MES regulator. **(A)** Schematic of PG-selective RNAi screen design. In “step 1”, *UAS-RNAi* males against each 703 candidate gene are crossed with females carrying two copies of *phm>mCherry*.*nls* to obtain F1 animals in which target gene is knocked down selectively in the PG. *Octβ3R* and *tor* were included in candidate gene sets as positive control showing L3 arrest phenotype. In “step 2”, PGs of RNAi animals, in which developmental defect was confirmed, were observed by histochemistry to investigate their cell number and the DNA content. **(B)** Pie chart showing the distribution of the phenotypic categories in step 1. The number of RNAi lines showing each phenotype is shown in the legend of each pie. NOP, no obvious phenotype. **(C)** Schematic of developmental transition of the PG cell number and the DNA content. PG cells at L1 stage (indicated as “Early PG cells”) undergo mitotic cell cycle to increase their number, and subsequent MES initiates endocycling which leads to mature PG cells with higher DNA content (indicated as “Late PG cells”). When MES regulator was inhibited in the PG, the PG cell number was increased and an increase in the DNA content was blocked (indicated as “Defects in MES”). Defects in endocycle progression results in decrease in the DNA content, whereas inhibition of downstream effector of endocycle should display no significant decrease in the DNA content. **(D)** Dot-plots showing the mean values of the cell number and the DNA content in the PG of control and RNAi animals. Each plot shows the average values of triplicate data sets in each group. Green and black plots indicate control animals which were cultured in standard culture medium and nutrient-rich German food (GF), respectively. Numbers indicate time points for sampling of control animals cultured in GF. All other data were obtained at day 6 after crossing in standard culture medium, including control shown by green plot. Purple and magenta plots show RNAi animals in which the PG cell number was significantly increased compared to the controls (*P* < 0.05; Dunnett’s multiple comparisons test). Magenta indicates RNAi against *cct* subunit genes.

To further dissect the regulatory mechanisms of MES, we performed a genetic screen using the Gal4/UAS system and RNAi. A previous study carried out a genome-wide PG-selective RNAi screen and identified 701 genes whose knockdown cause L3 or L1/L2/L3 arrest (630 and 71 genes, respectively) [26]. We therefore focused on these genes as novel MES regulator candidates and knocked them down in the PG to observe their potential effects on developmental transitions as well as the cell number and the DNA content in the PG (Fig 4A). In our screen, females carrying two copies of *phm>mCherry*.*nls* were crossed with *UAS-RNAi* males to drive a dsRNA or shRNA construct selectively in the PG of their offspring. In addition, RNAi lines not used in a previous genome-wide RNAi screen were used whenever available to exclude potential off-target effects (see S3 Table). Because knockdown of *target of rapamycin* (*tor*) and *β3-octopamine receptor* (*Octβ3R*) in the PG causes an L3 arrest phenotype [22, 27], these two genes were used as positive controls (Fig 4A). In this screen, we used standard cornmeal/yeast *Drosophila* culture medium, whereas all other experiments in this study were performed using nutrient-rich German Food (GF).

In the first step analyzing the developmental phenotype (indicated as ‘Step 1’ in Fig 4A), a larval arrest phenotype was confirmed in 442 genes; more specifically, L1/L2, L1/L2/L3 and L3 arrest were observed in 77 (11%), 18 (3%), and 347 genes (49%), respectively (Fig 4B). Other phenotypes, including delayed pupariation (7 genes) and embryonic lethality (1 gene), were observed in 8 genes (1%). In contrast, 253 genes (36%) showed no obvious phenotype (NOP; Fig 4B), which were excluded from further analysis. Next, we observed PG cells of 449 RNAi lines that showed either larval arrest or delayed pupariation (indicated as ‘Step 2’ in Fig 4A). The schematic diagram in Fig 4C shows the developmental change of the cell number and the DNA content in PG cells. Normal PG cells undergo mitotic cell cycle during the early larval stage (i.e. L1), then undergo MES and several rounds of endocycling to increase the DNA content during the L2 and L3 stages (Fig 4C). By contrast, as in the case of *fzr* RNAi, animals with MES-deficient PG are expected to be arrested at the larval stage with the PG showing an increased cell number and reduced DNA content (Fig 4C). In addition, it is expected that a defect in endocycle progression results in a reduced DNA content, whereas blocking the downstream pathway of endocycle does not cause a severe reduction in the DNA content (Fig 4C). Based on these criteria, the cell number and the DNA content were examined in the PG of each RNAi line using histochemistry at day 6 after crossing (Fig 4A). Fig 4D shows the mean value of the DNA intensity and the cell number in the PG of 449 RNAi lines, as well as the controls (*phm>mCherry*.*nls*) (green plot in Fig 4D, indicated as “Control” in the legend), raised on standard *Drosophila* medium. We also observed the PG of control animals cultured in nutrient-rich GF at 0, 24, 48, 72, and 96 hAH as a references (black plots in Fig 4D, indicated as “Control cultured on GF” in the legend). Of the 449 genes tested in Step 2, knockdown of 210 genes caused morphological or physiological defects in PG cells, including an abnormal distribution of DNA and nucleolus, apoptotic nuclear condensation, and a nontransparent cytoplasm (S3 Table, S6 Fig). The remaining 239 genes were statistically analyzed to reveal which RNAi animals showed a significant increase in the PG cell number compared with the controls, indicated by green plot in Fig 4D. With this analysis, we identified 31 genes whose knockdown caused a significant cell number increase in the PG (called “MES-related genes” hereafter) (magenta and purple plots in Fig 4D; summarized in S4 Table). To reveal biological processes important for MES, Gene ontology (GO)-term enrichment analysis was performed for MES-related genes, and we found that *chaperonin containing tcp1* (*cct*) genes were significantly enriched in the MES-related gene group. CCT proteins are subunits of the evolutionary conserved molecular chaperon complex, TRiC, which supports proper folding of cytoskeletal proteins and cell cycle regulators [28, 29]. Generally, TRiC is a hetero-oligomeric double-ring complex with eight subunits (CCT1–8) per ring [30]. Six *cct* subunit genes (*cct1*, *2*, *4*, *5*, *6*, and *8*) were included in 31 MES-related genes, and knockdown of these genes caused not only an increased cell number but also a severe reduction in the DNA content (magenta plots in Fig 4D). Taken together, our RNAi screen raised the possibility that TRiC is a novel MES regulator in the PG.

### TRiC is required for proper MES and endocycle progression in the PG

**Fig 5:**
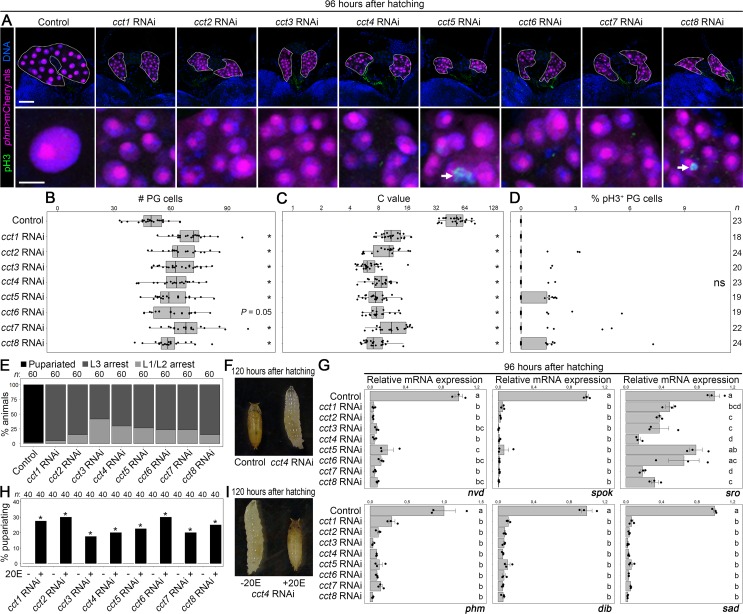
*cct* subunit genes are required for ecdysone biosynthesis in the PG. **(A)** The PG (upper panels) and PG cells in higher magnification (lower panels) of the controls (*phm>mCherry*.*nls*) and RNAi against each *cct* subunit genes (*phm>mCherry*.*nls cct-RNAi*) at 96 hAH. pH3 and DNA were detected by anti-pH3 antibody (green) and Hoechst (blue), respectively, and the nuclei of PG cells were labelled by mCherry.nls (magenta). The PGs are indicated by dotted lines. The arrows indicate pH3-positive PG cells. Scale bars: 50 μm (upper panels) and 10 μm (lower panels). **(B–D)** Scatter and box plots showing the cell number (B), the C value (C), and the percentage of pH3-positive cells (D) in the PG of control and *cct1-8* RNAi animals at 96 hAH. The asterisks indicate statistically significant differences between the control and *cct* RNAi (*P* < 0.05; Steel–Dwass test). *P*-values for all pairwise comparisons are shown in S1 Table. ns, not significant (*P* > 0.05). Box and dot plots as in Fig 1C–1E. The mean C value of control at 96 hAH was set to 53 (see S1B Fig). Sample sizes (the number of PGs) are shown in right side of each column. **(E)** Percentages of pupariated and L1/L2- and L3-arrested animals in the controls (*phm>+*) and *cct1–8* RNAi (*phm>cct-RNAi*) are shown. Sample sizes (the number of animals) are shown above each column. **(F)** Pupariated control and *cct4* RNAi arrested at the L3 stage. **(G)** The expression level of ecdysone biosynthetic genes was measured using qPCR. Scatter plots with average values of triplicate data sets are shown with SE (ten to fifteen larvae were pooled in each datum). Different lowercase letters indicate statistically significant differences (*P* < 0.05; Tukey’s multiple comparison test; see S1 Table). **(H)** Percentages of pupariated *cct1–8* RNAi animals, cultured on the medium with 20E (0.5 mg/g) or without 20E from 48 hAH are shown. The asterisks indicate statistically significant differences between 20E-fed and no 20E groups (*P* < 0.05; Fisher’s test). Sample sizes (the number of animals) are shown above each column. **(I)** *cct4* RNAi larva fed on -20E medium and pupariated *cct4* RNAi animal fed on +20E medium.

To investigate the role of TRiC in the PG, each *cct* subunit gene was knocked down in the PG. In contrast to the controls (*phm>mCherry*.*nls*) whose cell number and C value in the PG were around 50 and 53, respectively (Fig 5A–5C, S1A and S1B Fig), the PG cell number of *cct1*–*8* RNAi animals (*phm>mCherry*.*nls cct-RNAi*) reached 60–70, and their C value was around 8 at 96 hAH (Fig 5A–5C). These results indicate that *cct* subunit genes are required for proper MES. In addition, pH3 expression was detected in some, but not all, PGs of *cct* RNAi larvae (Fig 5D), but we could not observe statistically significant difference in pH3 expression between control and *cct* RNAi (Fig 5D). This suggests that *cct* genes are also required for proper progression of mitotic cell cycle. We next confirmed that development was mainly arrested at the L3 stage in *cct* RNAi animals (*phm>cct-RNAi*) (Fig 5E and 5F), that ecdysteroidogenic gene expression was significantly reduced in *cct* RNAi (Fig 5G), and that 20E administration restored larval-to-pupal transition in 20%–30% of animals (Fig 5H and 5I). The explanation for why only 20%–30% of *cct* RNAi was rescued is that the 20E concentration used in this rescue experiment may have been too high to trigger proper pupariation in *cct* RNAi animals. To test this possibility, we have administrated 20E against *cct8* RNAi animals at a concentration of 0.5 mg/g (used mainly in this paper), 0.05 mg/g, 0.005 mg/g, 0.0005 mg/g, and 0 mg/g. As S7 Fig shows, approximately 30% of *cct8* RNAi larvae fed on the medium with 0.5 and 0.05 mg/g of 20E undergo pupariation, but there was no significant difference in timing and the percentage of pupariation between these two groups. This suggests that lower efficiency of this rescue experiment is not explained by the concentration of 20E used. Considering that PG produces other humoral factors, including monoamines [27], one potential mechanism is that knockdown of *ccts* perturbs production of hormones other than ecdysone, which may cause developmental defects. Overall, these results indicate that TRiC is required for ecdysone biosynthesis in the PG to induce the larval-to-pupal transition.

**Fig 6:**
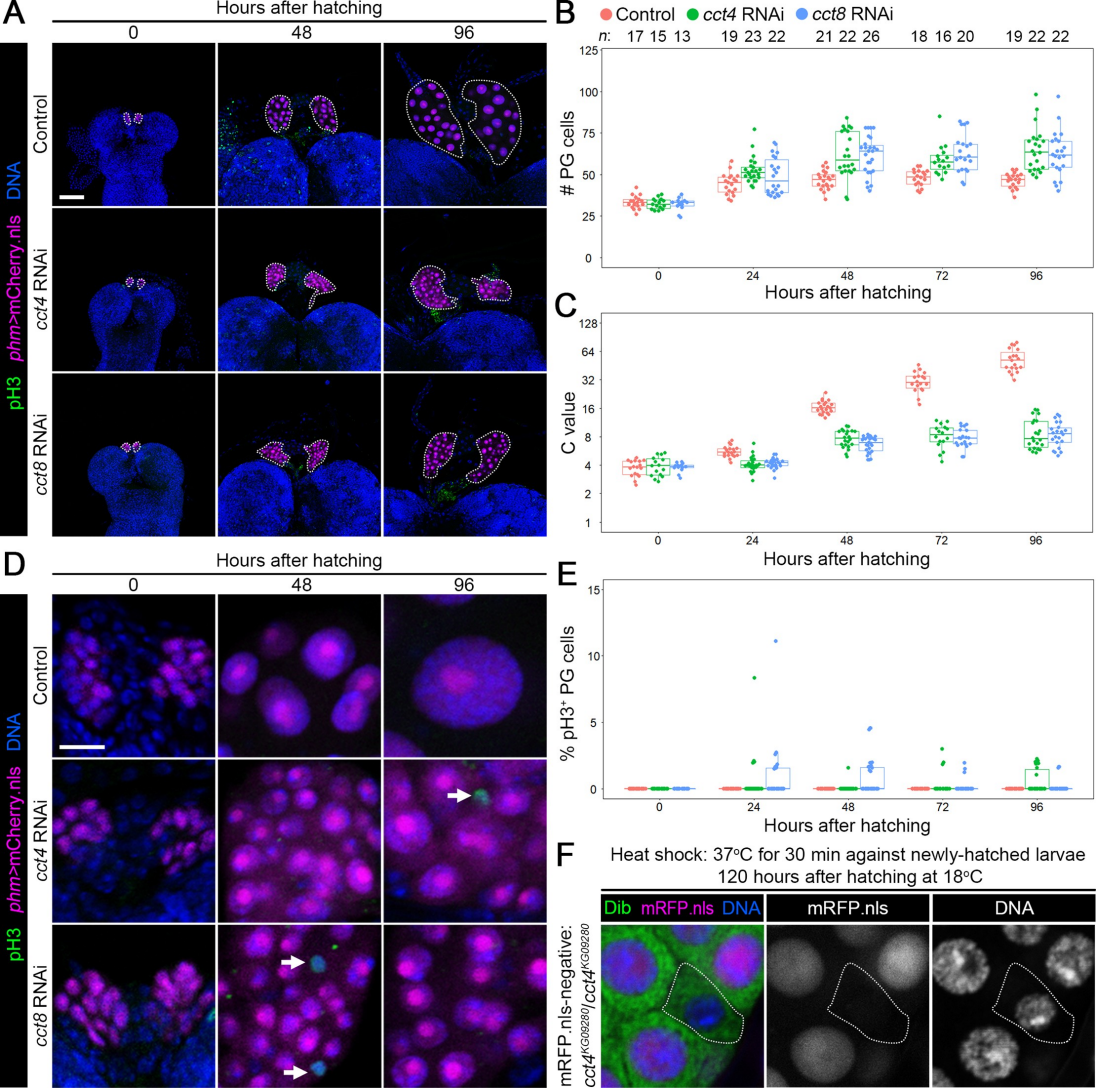
TRiC is required for proper progression of MES and endocycle in the PG. **(A)** The PG of the controls (*phm>mCherry*.*nls*), *cct4* RNAi (*phm>mCherry*.*nls cct4-RNAi*), and *cct8* RNAi (*phm>mCherry*.*nls cct8-RNAi*) at indicated time points. pH3 and DNA were detected by anti-pH3 antibody (green) and Hoechst (blue), respectively, and the nuclei of PG cells were labelled by mCherry.nls (magenta). The PGs are indicated by dotted lines. Scale bars: 50 μm. **(B and C)** Scatter and box plots showing the cell number (B) and the C value (C) in the PG of the controls (red), *cct4* RNAi (green), and *cct8* RNAi animals (blue) at indicated stages. *P*-values for all pairwise comparisons are shown in S1 Table (Steel–Dwass test). Box and dot plots as in Fig 1C–1E. The mean C value of control at 96 hAH was set to 53 (see S1B Fig), and all data points were normalized accordingly. Sample sizes (the number of PGs) are shown above each column. **(D)** PG cells in higher magnification of control, *cct4* RNAi, and *cct8* RNAi animals at indicated stages. The arrows indicate pH3-positive mitotic PG cells. Scale bars: 10 μm. **(E)** Scatter and box plots showing the percentage of pH3-positive PG cells of the controls (red), *cct4* RNAi (green), and *cct8* RNAi animals (blue) at indicated stages. *P*-values for all pairwise comparisons are shown in S1 Table (Steel–Dwass test). Sample sizes are the same as in B and C. **(F)** *cct4*^*KG092808*^/*cct4*^*KG092808*^ FLP-out clone induced by heat shock. Newly-hatched larvae are heat-shocked at 37°C for 30 min, and then recovered at 18°C until 120 hAH. *FRT40A* site was used in this clonal analysis. *cct4*^*KG092808*^ homozygous mutant cell (indicated by dotted line) is mRFP.nls-negative (magenta in left panel and middle panel). The PG and nuclear DNA were labelled by anti-Dib antibody (green in left panel) and Hoechst (blue in left panel and right panel), respectively. Scale bars: 10 μm.

Further observation of PG cells during larval development revealed that individual knockdown of *cct4* and *8* caused a delay in both the onset of the DNA content increase and cessation of the cell number increase: In the controls (*phm>mCherry*.*nls*), the PG cell number reached around 45 at 24 hAH and its C value was continuously elevated after 24 hAH (Fig 6A–6C). By contrast, in *cct4* and *8* RNAi animals (*phm>mCherry*.*nls cct4/8-RNAi*), the PG cell number reached around 60 by 48 hAH, and the C value of their PG cells did not increase after 48 hAH (Fig 6A–6C). Consistently, pH3-positive cells were detected in the PGs of *cct4* and *8* RNAi animals even after 24 hAH, although not statistically significant (Fig 6D and 6E). These results indicate that mitotic cell cycle is prolonged (i.e., MES is delayed) in the PG of *cct* RNAi. Furthermore, we found that the rate of the DNA content increase was suppressed in the PGs of *cct4* and *8* RNAi animals even after cessation of the cell number increase (Fig 6C). This result indicates that TRiC also regulates endocycle progression in the PG, as well as mitotic cell cycle and MES. To confirm this possibility, the *cct4* mutant line named *cct4*^*KG09280*^, carrying a P-element insertion on the *cct4* coding region that causes loss of *cct4* mRNA expression and developmental arrest at the L1/L2 stages (S8 Fig), was used for FLP/FRT-based clonal analysis. FLP-out clones carrying a *cct4*^*KG09280*^ homozygous mutation in the PG showed decreased DNA content (Fig 6F), confirming the importance of TRiC in endocycle progression.

### TRiC controls nuclear translocation of Fzr and downregulation of CycA

**Fig 7:**
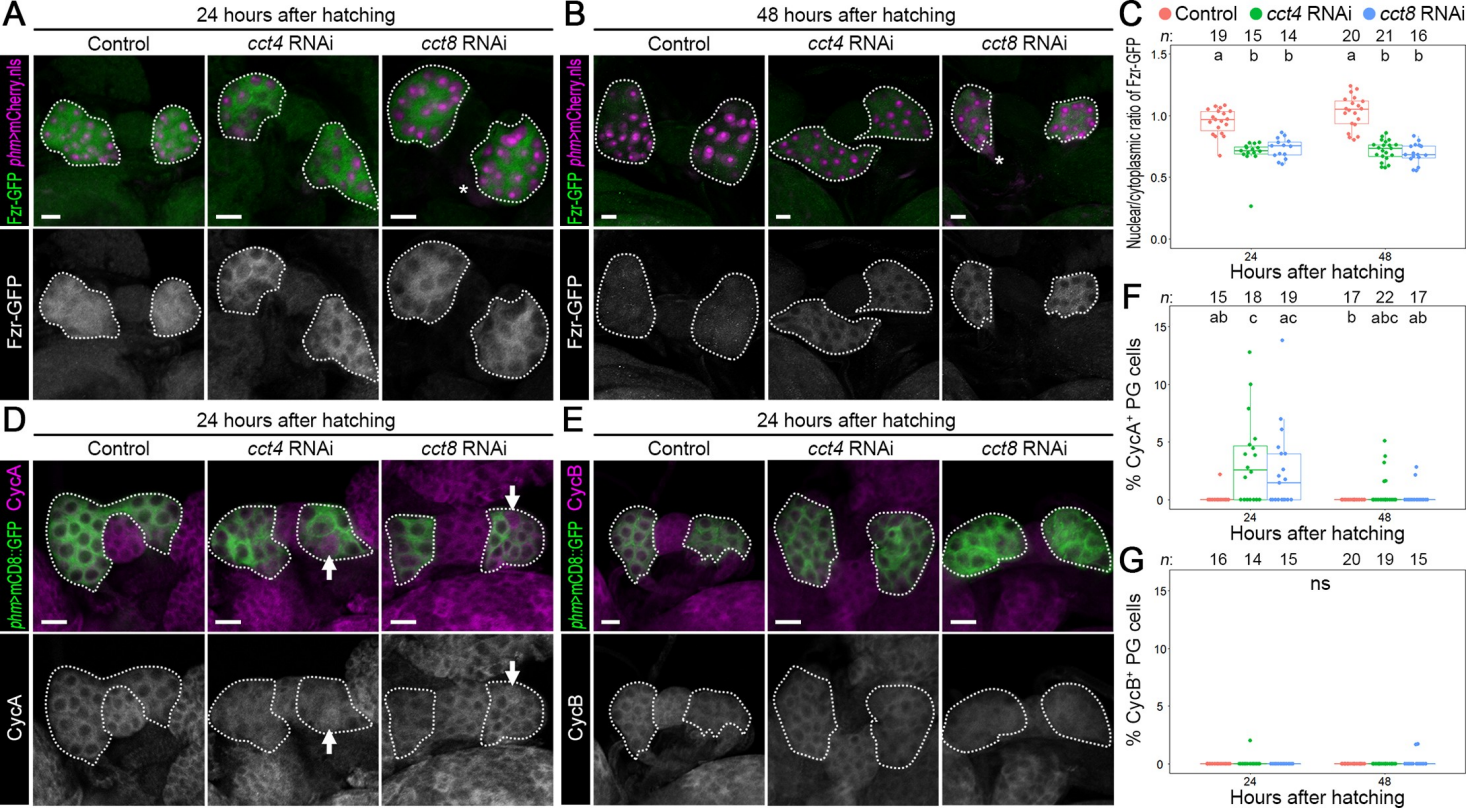
TRiC regulates expression of Fzr and mitotic cyclin in the PG. **(A and B)** Fzr-GFP expression (green and white in the upper and lower panels, respectively) was observed in the controls (*phm>mCherry*.*nls*, *fzr-GFP*), *cct4* RNAi (*phm>mCherry*.*nls cct4-RNAi*, *fzr-GFP*), and *cct8* RNAi (*phm>mCherry*.*nls cct8-RNAi*, *fzr-GFP*) using anti-GFP antibody at 24 (A) and 48 hAH (B). The nuclei of PG cells are labeled by mCherry.nls (magenta in the upper panels). The PGs are indicated by dotted lines. The asterisks indicate CC cells expressing 3xP3-dsRed marker in Fzr-GFP transgene. Scale bars: 10 μm. **(C)** Scatter and box plots showing the nuclear/cytoplasmic ratio of Fzr-GFP expression of the controls (red), *cct4* RNAi (green), and *cct8* RNAi animals (blue) at indicated stages. Different lowercase letters indicate statistically significant differences (*P* < 0.05; Steel–Dwass test; see S1 Table). Sample sizes (the number of PGs) are shown above each column. **(D and E)** CycA (D) and B expression (E) in the PG of the controls (*phm>mCD8*::*GFP*), *cct4* RNAi (*phm>mCD8*::*GFP cct4-RNAi*), and *cct8* RNAi animals (*phm>mCD8*::*GFP cct8-RNAi*) at 24 hAH. PG cells were labelled by mCD8::GFP (green in the upper panels), and CycA and B were detected by specific antibodies to each cyclin (magenta and white in the upper and lower panels, respectively). The PGs are indicated by dotted lines. The arrows indicate PG cells expressing CycA. Scale bars = 10 μm. **(F and G)** Scatter and box plots showing the percentage of CycA-positive (F) and CycB-positive PG cells (G) of the controls (red), *cct4* RNAi (green), and *cct8* RNAi animals (blue) at indicated stages. Different lowercase letters indicate statistically significant differences (*P* < 0.05; Steel–Dwass test; see S1 Table). ns, not significant. Sample sizes (the number of PGs) are shown above each column.

The above observations raised the question of how TRiC regulates MES and endocycling. To investigate whether TRiC controls MES via Fzr and mitotic cyclin regulation, expression of Fzr-GFP and mitotic cyclins was observed in the PGs of *cct4* and *8* RNAi animals. In the controls (*phm>mCherry*.*nls*, *fzr-GFP*), Fzr-GFP was detected in both the cytoplasm and nuclei of PG cells (Fig 7A–7C). In contrast, localization of Fzr-GFP into the nuclei was suppressed in the PGs of *cct4* and *8* RNAi larvae (*phm>mCherry*.*nls cct4/8-RNAi*, *fzr-GFP*) at 24 and 48 hAH (Fig 7A–7C), suggesting that TRiC regulates nuclear translocation of Fzr in the PG. Furthermore, probably due to the misregulation of Fzr nuclear translocation, CycA expression in the PG of *cct4* RNAi (*phm>mCD8*::*GFP cct4-RNAi*) was significantly increased than those in the controls (*phm>mCD8*::*GFP*) at 24 hAH (Fig 7D and 7F). CycA was also detectable in the PG of *cct8* RNAi (*phm>mCD8*::*GFP cct8-RNAi*) although this was not statistically significant ((Fig 7D and 7F). However, we could not observe enhanced CycB expression in *cct4* and *8* RNAi (Fig 7E and 7G). These results suggest that TRiC-deficient PG cells cannot undergo proper MES owing to CycA upregulation. To test this possibility, we investigated whether knockdown of *cycA* restores MES in *cct* RNAi animals. As Figs 5 and 6 show, the PG cell number in *cct4* and *8* RNAi larvae was around 60 at 96 hAH (S9A and S9B Fig), whereas the PG cell number was reduced to 40 when *cycA* RNAi was introduced into *cct4/8* RNAi (*phm>mCherry*.*nls cct4/8-RNAi cycA-RNAi*: referred to hereafter as *cct4/8 RNAi + cycA RNAi*) (S9A and S9B Fig). In addition, pH3-positive cells were not detectable in the PGs of *cct4/8* RNAi + *cycA* RNAi animals (S9A and S9D Fig). These observations indicate that TRiC-mediated downregulation of CycA is required for proper MES in the PG.

Moreover, knockdown of *cycA* in the PGs of *cct4/8* RNAi animals resulted in a DNA content increase to 16C (S9A and S9C Fig), indicating that TRiC-mediated CycA downregulation promotes endocycle up to 16C. However, *cycA* knockdown was not sufficient to restore the third round of endocycle, from 16 to 32C (S9C Fig), which is required for upregulation of ecdysone biosynthesis in the PG. As a result, developmental arrest was not rescued in *cct4/8* RNAi + *cycA* RNAi animals (without *UAS-mCherry*.*nls*: *phm>cct4/8-RNAi cycA-RNAi*) (S9E Fig). These results suggest that downregulation of CycA is not sufficient to rescue rounds of endocycle completely in the PG of *cct* RNAi.

**Fig 8:**
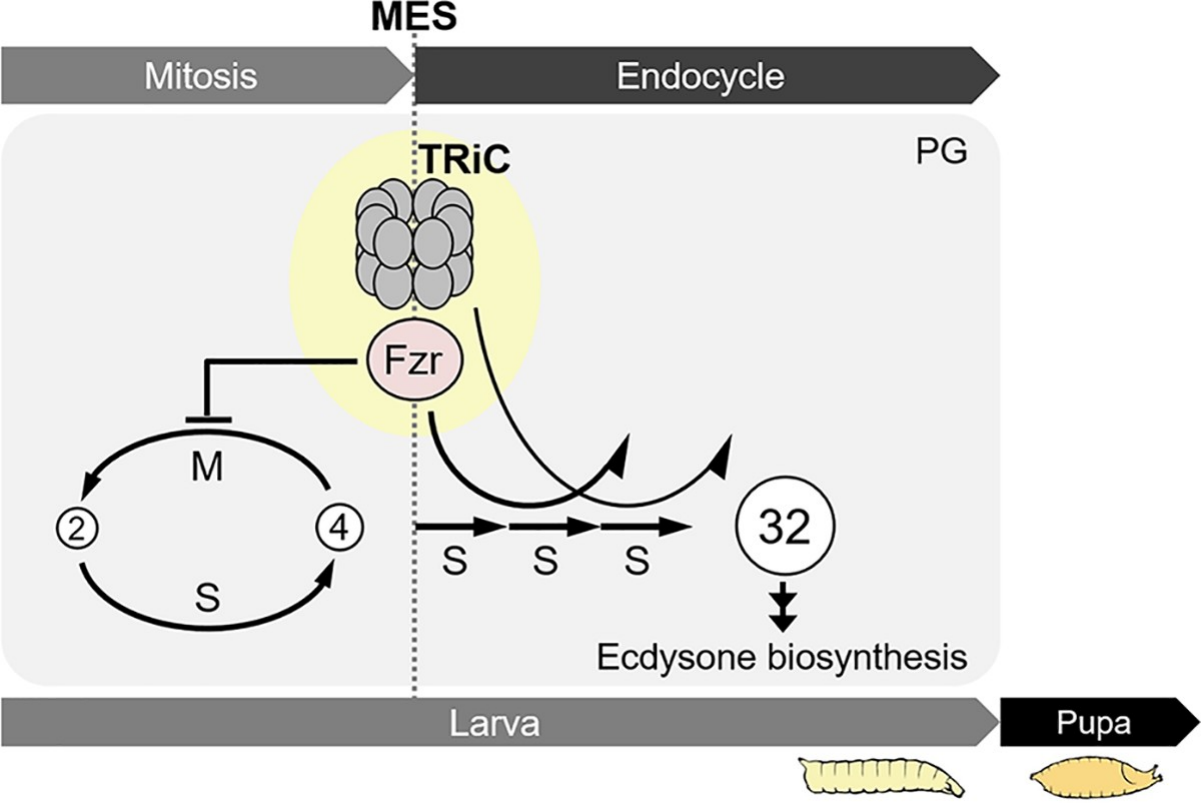
Current working model for TRiC-mediated MES regulation in the PG PG cells undergo MES in Fzr-dependent manner. Molecular chaperonin TRiC downregulates CycA at least in part by regulating Fzr nuclear translocation to induce MES and subsequent endocycling. Proper progression of MES and endocycle leads to the terminal differentiation of the PG, activation of ecdysone biosynthesis.

Based on our findings, here we propose a working model of TRiC-mediated control of MES and endocycle progression: TRiC downregulates CycA by regulating Fzr nuclear translocation to promote MES and endocycling (Fig 8).

## Discussion

MES is an essential cellular process that changes cell states from proliferation to growth and initiates terminal differentiation in multicellular organisms. Here we used the *Drosophil*a steroidogenic organ PG to study regulatory mechanisms of MES and found that PG cells undergo MES in a Fzr-dependent manner to activate ecdysteroid biosynthesis. Furthermore, our RNAi screen identified the evolutionary conserved chaperonin TRiC as a novel regulator of MES and endocycle progression. Further genetic analysis showed that TRiC downregulates CycA at least in part by regulating Fzr nuclear translocation to induce MES and subsequent endocycling. Based on these results, we propose that TRiC-mediated protein quality control is a fundamental mechanism supporting MES and subsequent endocycling that promotes terminal differentiation.

### TRiC-mediated regulation of MES and endocycle

We investigated the role of TRiC in regulating Fzr and mitotic cyclin expression in the PG, and found that TRiC is required for nuclear translocation of Fzr (Fig 7). This result suggests that TRiC supports Fzr folding to facilitate its translocation into the nuclei. Furthermore, knockdown of *cct* subunit gene resulted in increased CycA expression (Fig 7), and knockdown of *cycA* along with *cct* subunit genes prevented PG cell number increase (S9 Fig), indicating that TRiC promotes CycA inactivation, which allows PG cells to undergo MES. Because nuclear translocation of Fzr was blocked in *cct* RNAi, increased CycA expression in the nuclei of *cct* RNAi is likely due to decreased Fzr translocation into the PG cell nuclei. However, in contrast to CycA, CycB expression was not disturbed in *cct* subunit RNAi (Fig 7). Thus, we speculate that TRiC is unnecessary for Fzr to recognize CycB.

Although the PG cell number was significantly increased in *cct* RNAi, we could not observe a statistically significant increase in the percentage of pH3-positive PG cells in *cct* RNAi (Figs 5 and 6). The explanation for why pH3 was not detected frequently is that *cct* is also required for proper progression of mitotic cell cycle in the PG. Indeed, the PG cell number was not continuously increased in *cct* RNAi (Fig 6). Actually, it has been reported that TRiC regulates the disassembly of mitotic checkpoint complex [31] and mitotic cell cycle events such as sister chromatid separation [32]. Further elucidation of the regulatory mechanism of TRiC-mediated mitotic cell cycle is an important step to understanding the role of TRiC in cell cycle control.

In addition to the importance of TRiC in MES, we revealed that TRiC also has a critical role in regulating endocycle progression: inhibition of *cct* subunit genes caused endocycle arrest at around 8C (Figs 5 and 6), and knockdown of *cycA* together with *cct* in the PG partially restored endocycle up to 16C (S9 Fig). These results indicate that TRiC-mediated CycA downregulation is also required for progression of endocycling in the PG, perhaps due to reduced nuclear translocation of Fzr. Indeed, it has been reported that CycA as well as Fzr controls endocycle progression in the *Drosophila* [33–35]. However, *cycA* knockdown was not enough to restore the third round of endocycle in the PG of *cct* RNAi animals (S9 Fig). This suggests that other downstream factor(s) of TRiC or Fzr promote the third endocycle independently of CycA downregulation. Because Fzr also regulates Geminin degradation, a DNA replication inhibitor, to promote endocycle progression [33], one possible mechanism is that Fzr facilitates entry into the S-phase through suppression of Geminin to execute proper progression of endocycle in the PG. Furthermore, given that TRiC supports numerous proteins’ folding, including tubulin and actin [28, 29], we propose that TRiC-mediated protein quality control is a fundamental mechanism supporting MES and subsequent endocycling, which leads to the terminal differentiation.

### PG is suitable for studying regulatory mechanisms of MES and endocycle

In this study, we used the PG as a model organ to study MES and endocycle regulatory mechanisms because of the organ’s simple structure and the correlation between endocycling and ecdysone biosynthesis. PG cells undergo MES at the end of L1 and carry out repeated rounds, at least three times, of endocycle during the L2 and L3, which is essential for activation of ecdysone biosynthesis (Figs 1–3, S1 and S4 Figs). In contrast to PG cells, CA cells do not seem to undergo mitotic cell cycle and perform only one round of endocycle during the larval stage (Fig 1 and S2 Fig). These two types of endocrine cells originate from homologous ectodermal cells, and homeobox (Hox) gene expression controls PG and CA specification during embryogenesis [36]. Because CA and PG cells originate from deformed (Dfd)- and sex comb-reduced (Scr)-positive ectoderms, respectively [36], one possible mechanism is that distinct downstream genetic programs induced by Dfd and Scr determine the timing of MES and the activity of endocycle in these cells.

Our PG-selective RNAi screening identified not only the *cct* subunit genes required for proper MES but also other MES-related genes (summarized in S3 Table). Considering that enhancement of *fzr* transcription is a common step to triggering MES in *Drosophila* and other organisms [1, 10–18], our results will provide a solid basis for further investigating regulatory mechanisms of how *fzr* expression is upregulated to initiate MES. In *Drosophila* ovarian follicle cells, for example, the Notch signaling pathway promotes *fzr* transcription during the MES period [10, 11]. However, because core components of Notch signaling were not included in our list of MES-related genes (S3 Table), the regulatory mechanisms of MES seem to be distinct between the PG and follicle cells. Thus, other signaling pathways are likely involved in transcriptional regulation of *fzr* in the PG. Furthermore, we identified a group of genes whose knockdown causes a significant decrease in the DNA content (Fig 4 and S3 Table), suggesting that these genes regulate endocycle progression. Endocycling in the PG is controlled by nutrient signaling, including the insulin/TOR signaling pathway regulating the third endocycle [22]. However, upstream signaling pathways of the first, second, and fourth endocycle, as well as MES, have not been identified. Thus, detailed and systematic analysis of both MES-related and endocycle-related genes will shed light on the molecular mechanisms of how environmental and genetic cues are integrated into MES and endocycle progression in the PG to determine the onset of ecdysone biosynthesis.

In summary, we have demonstrated the genetic evidence showing the importance of TRiC in the regulation of MES and endocycle. Considering that TRiC suppresses accumulation of mitotic cyclins through the generation of functional Cdh1 protein in yeast [32], we propose that TRiC-mediated regulation of Cdh1/Fzr is an evolutionary conserved mechanism that promotes exit from mitotic cell cycle, including MES. Moreover, several lines of evidence have shown that some *cct* subunit genes are involved in the survival and proliferation of cancer cells [37]. Considering that cancer tissues possess endocycling cells at a high frequency and that endocycle is considered crucial for tumorigenesis [6–8], elucidating the role of TRiC in MES and endocycle progression is a fundamental step to revealing TRiC-mediated control of oncogenesis. This study thus provides a solid basis for revealing genetic programs that control initiation and progression of endocycle.

## Materials and methods

### *Drosophila* stocks

Genotypes of the flies used in this study are summarized in S5–S7 Tables, and *UAS-RNAi* lines used for PG-selective RNAi screen and its phenotype were summarized in S3 Table. Fly stocks were maintained on standard *Drosophila* cornmeal/yeast medium at 18 or 25°C under a 12-hour light/dark cycle.

### Analysis of developmental progression

To obtain larvae just after hatching, parent flies were maintained in the bottle and allowed to lay eggs for 24 hours on grape juice agar plates supplemented with yeast powder. Newly hatched larvae were transferred to vials with nutrient-rich medium named as “German food (GF)” (https://bdsc.indiana.edu/information/recipes/germanfood.html). Larvae were cultured at 25°C under a 12-hour light/dark cycle, and developmental stages and lethality were scored periodically.

### Quantitative RT-PCR (qPCR)

Total RNA was extracted from whole larvae using TRIzol (Thermo). Reverse-transcription was performed using SuperScript III (Invitrogen). cDNA was used as a template for qPCR using Quantifast SYBR Green PCR kit (QIAGEN) and Rotor-Gene Q (QIAGEN). The expression level of target gene was normalized using an endogenous control, *ribosomal protein 49* (*rp49*), and the relative expression level was calculated (relative expression level = expression value of the gene of interest/expression value of *rp49*). Primer sets used for qPCR are shown in S8 Table.

### Ecdysteroid measurement

Ten larvae were rinsed with distilled water, and collected in a 1.5 ml microcentrifuge tube. The larvae were homogenized in 400 μl of methanol with a plastic pestle at room temperature. The samples were centrifuged at 15,000 g for 5 min at 4°C, and 60 μl of the supernatant (equivalent to 1.5 larvae) was subjected to vacuum desiccation. Dried extract was re-dissolved in 50 μl of EIA buffer (Cayman Chemical). Ecdysteroid was quantitated by enzyme-linked immunosorbent assay (ELISA) using 20E EIA antiserum, 20E AchE tracer, and Ellman’s reagent (Cayman Chemical) according to manufacturer’s protocol. Standard 20E was purchased from Sigma.

### 20E administration

To rescue developmental arrest in RNAi animals, larvae were transferred to GF with 0.5 mg/g 20E at 48 hAH. Larvae transferred to GF without 20E at the same time point were used as control. Developmental stages were scored at 24-hour intervals.

### Immunostaining and histochemistry

Larvae were dissected in phosphate buffered saline (PBS) and fixed for 25 min with 4% paraformaldehyde (PFA) in 0.1% PBT (0.1% Triton X-100 in PBS). Tissues were washed with 0.1% PBT three times for 10 min each, permeabilized with 1% PBT (1% Triton X-100 in PBS) for 5 min, blocked with 2% goat serum (Sigma, G9023) in 0.1% PBT for 30 min, and then incubated at 4°C overnight with primary antibodies diluted in blocking solution. Tissues were washed with 0.1% PBT three times for 10 min each, and incubated at 4°C overnight with Alexa 488- or Alexa 546-conjugated secondary antibodies (Thermo) in 0.1% PBT. Together with the secondary antibody, Hoechst 33342 (Thermo, H3570) was added at a 1:1500 dilution to detect DNA. After washing with 0.1% PBT three times for 10 min each, tissues were mounted in mounting medium.

Fly-FUCCI probe expressed in the PG was observed as follows: Larvae were dissected in phosphate buffered saline (PBS) and fixed for 25 min with 4% PFA in 0.1% PBT; Tissues were washed with 0.1% PBT three times for 10 min each, and incubated with Hoechst at a 1:1500 dilution at 4°C overnight; We did use neither anti-GFP nor anti-mRFP antibody; After washing with 0.1% PBT three times for 10 min each, tissues were mounted in mounting medium.

The following primary antibodies were used at indicated dilutions: rabbit polyclonal anti-Dib (a gift from M. B. O’Connor), 1:500; guinea pig polyclonal anti-Sro (a gift from R. Niwa), 1:500; rabbit polyclonal anti-pH3 (Merck, 06–570), 1:500; mouse monoclonal anti-CycA (DSHB, A12), 1:25; mouse monoclonal anti-CycB (DSHB, F2F4), 1:25; mouse monoclonal anti-GFP (Thermo, A11120), 1:1000; and chicken polyclonal anti-GFP (Abcam, ab13970), 1:500.

Images were taken with a Zeiss LSM700, and the pictures’ properties including the cell number and Fzr-GFP expression level were analyzed using Image J/Fiji [38]. Cell counting was performed using the plugin named Cell Counter. Fzr-GFP signal intensity in the PG was obtained from stacked slices of PG, and normalized by the signal intensity in the CA. Measurement of the DNA content was performed as described below.

### Ploidy measurements and DNA quantification

Ploidy measurement was performed as previously described [39] with minor modifications. The C value in the PG and CA was determined using the following methods. Larvae and adult male flies were dissected in 0.7% NaCl. Brain-ring gland complex and testes were treated with 0.5% sodium acetate for 10 min and then fixed for 30 min with 4% paraformaldehyde in 0.1% PBT. After washing twice with 0.1% PBT, tissues were squashed on an APS-coated slide and submerged in liquid nitrogen to remove coverslip. Tissues were dehydrated in ethanol for 15 min and washed with PBS three times for 10 min each. Slides were stained with Hoechst (1:5000) for 15 min, washed with PBS three times for 10 min each, and mounted in mounting medium. Testes within each slides were imaged at the same gain and settings, in order to use the sperm cells as an internal control. Images were taken with a Zeiss LSM700, and analyzed using Image J/Fiji [38]. Regions were drawn around each nucleus using the trace function and the fluorescence intensity was measured within each region. The mean DNA staining intensity of sperm cells (1C) on the same slide was analyzed and set to 1. The average intensities of PGs were calculated on the basis of the C value in sperm cells.

To measure and quantify DNA signal intensity in the PG, a series of images obtained by immunostaining/histochemistry were processed using Image J/Fiji as follows. DNA signal overlapped with binarized and filled Sro signal (*Oregon R*) or *phm>*mCherry.nls signal (transgenic lines) was obtained using a function named “Image Calculator”. Processed images were stacked, DNA signal in the PG was drawn around their nuclei using the trace function, and the fluorescent intensity was measured within the region. DNA staining intensity in the PG was adjusted using average DNA staining intensity obtained from z-stacked images of the brain lobe. Normalized DNA staining intensity was further divided by the PG cell number to obtain mean DNA intensity per PG cell. In accordance with the result of ploidy measurement, the mean C value in the PG of *Oregon R* at 84 hAH was set to 58 (see Fig 1B and 1C), and the mean C values in the PG of *phm>mCherry*.*nls* and *phm>dicer2 mCherry*.*nls* at 96 hAH were set to 53 and 54, respectively, (see S1A and S1B Fig).

### BrdU incorporation

Larvae were dissected in Ringer’s solution, and the tissues were incubated for 30 min with 100 mM BrdU (Sigma, B5002) diluted in Ringer’s solution and then fixed in 4% PFA for 20 min. Fixed tissues were briefly washed twice in 0.01% PBT (0.01% Triton X-100 in PBS), washed in 0.1% PBT twice for 10 min each, and treated with 2N HCl for 30 min. The tissues were briefly washed twice in 0.01% PBT, washed in 0.1% PBT twice for 10 min, and incubated with blocking solution for 30 min. The tissues were incubated with primary antibody against BrdU diluted 1:20 in blocking solution overnight at 4°C. The tissues were washed in 0.1% PBT for 10 min three times, and incubated 4°C overnight with Alexa 488 fluor-conjugated secondary antibody (mouse IgG, Thermo Fisher, A-11001) and Hoechst diluted at 1:1000 and 1:1500, respectively, in 0.1% PBT. The tissues were washed in 0.1% PBT for 10 min three times and mounted on a slide glass with mounting medium. Images were taken with a Zeiss LSM700, and their analysis was performed using Image J/Fiji [38].

### PG-selective RNAi screening

Ten virgins carrying two copies of PG-selective *phm-Gal4* and *UAS-mCherry*.*nls* were crossed with five *UAS-RNAi* males to obtain the offspring in which gene of interest is knocked down in the PG. Parent flies were cultured on standard *Drosophila* medium in plastic vials for 2 days. Developmental phenotype of their progenies was observed to confirm developmental defects at day 10 after crossing. The PG of RNAi animals showing developmental defect were observed using histochemistry at day 6 after crossing as follows. Larvae at day 6, in which most of control larvae (*phm>mCherry*.*nls*) are in wandering stage, were dissected in PBS and fixed for 25 min with 4% PFA in 0.1% PBT. Tissues were washed with 0.1% PBT three times for 10 min each, and stained with Hoechst 33342 (1:1500 in 0.1% PBT) to observe the DNA content and distribution and morphology of PG cells. Images were taken with a Zeiss LSM700, and their analysis was performed using Image J/Fiji [38]. DNA quantification was performed as described above (see Ploidy measurement).

### Statistical analysis

Statistical analyses were performed using R (http://www.R-project.org/). Exact *P*-values of Steel Dwass and Tukey’s multiple comparison test in main and supplemental figures are shown in S1 and S2 Tables. All numerical data except for the data obtained in a RNAi screen are shown in S1 Data. Numerical data in the RNAi screen are shown in S3 Table. In our RNAi screen, a significant increase in the PG cell number was determined using Dunnet’s multiple comparison test. In all statistical analysis, *P* < 0.05 was considered to represent a statistically significant difference. GO-term enrichment analysis was performed to evaluate which biological process term is enriched in a group of genes using Reactome resource in PANTHER (http://pantherdb.org/).

## Supporting information

S1 Data Supporting data. (XLSX)

S1 Fig Cell cycle properties in PG cells. **(A)** Sperm cells of *Oregon R* (left panel) and PG cells at 96 hAH of control [*phm>mCherry*.*nls* (middle panel) and *phm>dicer2 mCherry*.*nls* (right panel)]. DNA was stained by Hoechst. Scale bars: 10 μm. **(B)** Scatter plots with mean value of relative signal intensity of Hoechst in *Oregon R* sperm cells and PG cells of control. The mean C value in sperm cells was normalized to 1, and accordingly, the mean values in PG cells of *phm>mCherry*.*nls* and *phm>dicer2 mCherry*.*nls* at 96 hAH were set to 53 and 54, respectively. Sample sizes (the number of sperm and PG cells) are shown above each column. **(C)** Incorporation of BrdU in the PG at 84 hAH. Incorporated BrdU was detected by anti-BrdU antibody (magenta in upper panel), the PG cell was stained using specific antibody against Dib (green in upper panel), and DNA was detected by Hoechst (blue in upper panel). The PG is indicated by dotted line. Early, middle (mid), and late-S phase cells were indicated by the arrow, arrowhead, and sharp arrowhead, respectively, and the zoomed images of these cells were shown in the lower panels. Scale bars: 50 μm (upper panel) and 10 μm (lower panels). **(D)** Scatter and box plots showing the percentage of early, mid, and late-S phase PG cells at 96 hAH. Different lowercase letters indicate statistically significant differences (*P* < 0.05; Steel–Dwass test, see S2 Table). Box and dot plots as in Fig 1C–1E. Sample sizes (the number of PGs) are shown above column. **(E and F)** Schematics of *Drosophila* FUCCI system. E2F1^1-230^-fused GFP (GFP.E2F1) and CycB^1-266^-fused mRFP1 (mRFP1.CycB) expressed under the control of Gal4/UAS system were degraded through CRL4- and APC/C-dependent manner, respectively (E). Since CRL4 and APC/C-dependent protein degradation are active at S and G1 phase in mitotic cell cycle, G1-, S-, and G2/M-phase cells were labelled by GFP, mRFP1, and both GFP and mRFP1, respectively (F). **(G)** The expression patterns of GFP.E2F1 (green and white in the upper and middle panels, respectively) and mRFP1.CycB (magenta and white in the upper and lower panels) in the PG of FUCCI reporter-expressing animals (*phm>GFP*.*E2F1 mRFP1*.*CycB*) at indicated time points. DNA was stained by Hoechst (blue in the upper panels). The PGs are indicated by dotted lines. The arrows indicate mitotic cell showing metaphase-like nuclear shape, which were counted as “mitotic cells” (yellow in H). Scale bars: 10 μm. **(H)** Scatter and box plots showing the percentage of GFP.E2F1-positive (green), mRFP1.CycB (red), both GFP.E2F1 and mRFP1.CycB-positive (orange), and mitotic cell (yellow) at indicated time points. *P*-values for all pairwise comparisons are shown in S2 Table (Steel–Dwass test). Sample sizes (the number of PGs) are shown above column.
(TIF)

S2 Fig CA cells undergo endocycle. **(A)** CycE expression in the CA. CycE was detected by anti-CycE antibody (green), and the CA was labeled by mCD8-fused RFP (mCD8::RFP; magenta) expressed under the control of *jhamt-Gal4*, at indicated time points. The CAs are indicated by dotted lines. Scale bars: 50 μm. **(B)** Scatter and box plots showing the percentage of CycE-positive CA cells at indicated stages. Different lowercase letters indicate statistically significant differences (*P* < 0.05; Steel–Dwass test; see S2 Table). Sample sizes (the number of CAs) are shown above each column. **(C)** Incorporation of BrdU in the CA. Incorporated BrdU was detected by anti-BrdU antibody (green), and the CA was labeled by mRFP (magenta) at indicated time points. The CAs are indicated by dotted lines. Scale bars: 50 μm. **(D)** Scatter and box plots showing the percentage of BrdU-positive CA cells at indicated stages. Different lowercase letters indicate statistically significant differences (*P* < 0.05; Steel–Dwass test; see S2 Table). Sample sizes (the number of CAs) are shown above each column.
(TIF)

S3 Fig Fzr and pH3 expression in the PG. **(A)** Fzr-GFP expression (green and white in the upper and lower panels, respectively) in the PG of the controls (*phm>dicer2 mCherry*.*nls*, *fzr-GFP*) and *fzr* RNAi animals (*phm>dicer2 mCherry*.*nls fzrRNAi*, *fzr-GFP*) at indicated time points. PG cells were labeled by mCherry.nls (magenta in the upper panels), and pH3 was stained by specific antibody to pH3 (blue in the upper panels). The PGs are indicated by dotted lines. The asterisks indicate CC cells expressing 3xP3-dsRed marker in Fzr-GFP transgene. Scale bars: 50 μm. **(B and C)** Scatter and box plots showing the relative expression of Fzr-GFP (B) and the percentage of pH3-positive cells (C) in the PG of the controls (red) and *fzr* RNAi (blue) at indicated stages. Different lowercase letters indicate statistically significant differences (*P* < 0.05; Steel–Dwass test; see S2 Table). Sample sizes (the number of PGs) are shown above each column.
(TIF)

S4 Fig *fzr* is required for ecdysone biosynthesis in the PG. **(A and B)** Percentages of L1, L2, L3, and pupariated animals in the controls (*phm>dicer2*) and *fzr* RNAi (*phm>dicer2 fzrRNAi*) during development. Sample sizes (the number of animals) are indicated in parentheses. **(C)** Pupariated control and *fzr* RNAi arrested at the L3 stage. **(D)** The expression level of ecdysone biosynthetic genes in the controls and *fzr* RNAi measured using qPCR at indicated time points. Average values of triplicate data sets with SE and scatter plots are shown. Ten to fifteen larvae were pooled in each datum. Different lowercase letters indicate statistically significant differences (*P* < 0.05; Tukey’s multiple comparison test; see S2 Table). **(E)** Whole-body ecdysteroid levels in the controls and *fzr* RNAi animals at 96 hAH measured using ELISA. Ecdysteroid levels of five independent data sets are shown by scatter and box plots. Ten larvae were pooled in each datum. The asterisk indicates statistically significant differences (*P* < 0.05; Welch’s two sample *t*-test). **(F)** Percentages of pupariated *fzr* RNAi animals cultured on the medium with 20E (0.5 mg/g) or without 20E from 48 hAH. Sample sizes (the number of animals) are indicated in parentheses. The asterisk indicates statistically significant differences (*P* < 0.05; Fisher’s test). **(G)** *fzr* RNAi larva fed on -20E medium and pupariated *fzr* RNAi animal fed on +20E medium.
(TIF)

S5 Fig CycA and B expression in the PG of *fzr* RNAi during development. CycA (A) and B expression (B) in the PG of the controls (*phm>dicer2 mCD8*::*GFP*) and *fzr* RNAi larvae (*phm>dicer2 mCD8*::*GFP fzr-RNAi*) at indicated time points. PG cells were labelled by mCD8::GFP (green in the upper panels) and CycA and B were detected by specific antibodies against each cyclin (magenta and white in the upper and lower panels, respectively). The PGs are indicated by dotted lines. Scale bars: 50 μm. The percentage of CycA and B-positive PG cells of control and *fzr* RNAi at 24, 48, 72, and 96 hAH is summarized in Fig 3C and 3D.
(TIF)

S6 Fig Morphological defects in PG cells observed in RNAi screen. **(A)** PG cells of the controls (*phm>mCherry*.*nls*) and RNAi animals (*phm>mCherry*.*nls gene-of-interest-RNAi*) showing morphological defects. Each phenotypic groups are categorized into “A”–”G” groups as indicated in lower comments. DNA was stained by Hoechst (blue and white in the upper and middle panels, respectively), and the nuclei of PG cells were labelled by mCherry.nls (magenta and white in the upper and lower panels, respectively). The arrows, arrowheads, and narrow arrowheads indicate interspace in the nuclei, duplicated nucleolar-like structure, and strong DNA puncta, respectively. Scale bar: 10 μm. **(B)** The PG of control and *cdsA* RNAi. The PG of *cdsA* RNAi is untransparent compared to control, which is categorized as “H” in this screening. The PGs are indicated by dotted lines. Scale bar: 50 μm. **(C)** Pie chart showing the distribution of the phenotypic categories of morphological defects in PG cells. Sample sizes (the number of animals) are indicated in parentheses.
(TIF)

S7 Fig 20E administration to *cct8* RNAi animals. The percentages of pupariated *cct8* RNAi animals, cultured on the medium with 20E (5 x 10^−4^, 5 x 10^−3^, 5 x 10^−2^, and 5 x 10^−1^ mg/g) or without 20E from 48 hAH, at indicated stages. Sample sizes (the number of animals) are indicated in parentheses. ns, not significant (Fisher’s test, *P* > 0.05).
(TIF)

S8 Fig Characterization of *cct4*^*KG09280*^ mutant. **(A)** Schematic diagram of *cct4* gene region and *KG09280* insertion site. The arrows indicate the primer sets used for qPCR to measure *cct4* expression level. **(B)** The relative expression level of *cct4* in wild-type (*+/+*) and *cct4*^*KG09280*^ homozygous mutant (*cct4*^*KG09280*^/*cct4*^*KG09280*^) measured using qPCR at 24 hAH. Average values of triplicate data sets with SE and scatter plots are shown. The asterisk indicates statistically significant differences (*P* < 0.05; Welch’s two sample *t*-test). **(C)** Agarose gel electrophoresis of PCR product of *rp49* and *cct4* in wild-type and *cct4*^*KG09280*^. **(D)** Percentages of pupariated and L1/L2- and L3-arrested animals in wild-type and *cct4*^*KG09280*^ heterozygous and homozygous mutant are shown. Sample size (the number of animals) are shown above each column. **(E)** Percentages of survival in wild-type and *cct4*^*KG09280*^ heterozygous and homozygous mutant are shown at indicated time points. Sample sizes are the same as in D.
(TIF)

S9 Fig Genetic interaction between *cct* subunits and *cycA*. **(A)** The PG (upper panels) and PG cells in higher magnification (lower panels) of *cct4* RNAi (*phm>mCherry*.*nls cct4-RNAi*), *cct4* RNAi + *cycA* RNAi (*phm>mCherry*.*nls cct4-RNAi cycA-RNAi*), *cct8* RNAi (*phm>mCherry*.*nls cct8-RNAi*), and *cct8* RNAi + *cycA* RNAi (*phm>mCherry*.*nls cct8-RNAi cycA-RNAi*) at 96 hAH. pH3 and DNA were detected by anti-pH3 antibody (green) and Hoechst (blue), respectively, and the nuclei of PG cells were labelled by mCherry.nls (magenta). The PGs are indicated by dotted lines. The arrows indicate pH3-positive PG cells. Scale bars: 50 μm (upper panels) and 10 μm (lower panels). **(B–D)** Scatter and box plots showing the cell number (B), the C value (C), and the percentage of pH3-positive cells (D) in the PG of *cct4* RNAi, *cct4* RNAi + *cycA* RNAi, *cct8* RNAi, and *cct8* RNAi + *cycA* RNAi animals at 96 hAH. The asterisks indicate statistically significant differences (*P* < 0.05; Mann–Whitney U test). Box and dot plots as in Fig 1C–1E. The mean C values of *cct4* and *cct8* RNAi at 96 hAH were set to 9 and 8.8, according to their mean C value at the same time point (Fig 6C). Sample size (the number of PGs) are shown above each column. **(E)** Percentages of pupariated and L1/L2- and L3-arrested animals in *cct4* RNAi, *cct4* RNAi + *cycA* RNAi, *cct8* RNAi, and *cct8* RNAi + *cycA* RNAi are shown. Sample sizes (the number of animals) are shown above each column. ns, not significant (*P* > 0.05; Fisher’s test).
(TIF)

S1 Table Exact *P*-value in main figures. * Steel Dwass multiple comparison test
** Tukey’s multiple comparison test
^a^ All data were obtained in *Oregon R*
^b^ All data were obtained in *phm>mCherry*.*nls*, *fzr-GFP*.
(XLSX)

S2 Table Exact *P*-value in supplemental figures. * Steel Dwass multiple comparison test
** Tukey’s multiple comparison test
^a^ All data were obtained in *Oregon R*
^c^ All data were obtained in *phm>GFP*.*E2F1 mRFP1*.*CycB*.
(XLSX)

S3 Table RNAi screen results. * BDSC, Bloomington Drosophila Stock Center; VDRC, Vienna Drosophila Resource Center; NIG, National Institute for Genetics; NIG TRiP, Transgenic RNAi Project (TRiP) in NIG.
** NOP, no obvious phenotype; Delayed P, delayed pupariation
*** Morphological or physiological defects in PG cells are classified into A–H (see S6 Fig).
^a^ Control cultured on the standard cornmeal/yeast medium, investigated at day 6 after crossing
^b^ Control cultured on GF, investigated at indicated time points.
(XLSX)

S4 Table MES-related genes. * *cct* subunit genes.
(XLSX)

S5 Table Fly stocks used in this study. * BDSC, Bloomington Drosophila Stock Center; VDRC, Vienna Drosophila Resource Center; Kyoto DGGR, Kyoto Drosophila Genomics and Genetic Resources; NIG, National Institute of Genetics
** Balancer carrying GFP was introduced in place of balancer without fluorescent marker.
(XLSX)

S6 Table *UAS-RNAi* lines used in this study, except in RNAi screen. * BDSC, Bloomington Drosophila Stock Center; VDRC, Vienna Drosophila Resource Center; NIG, National Institute of Genetics.
(XLSX)

S7 Table Genotypes of the flies used in this study. * *UAS-mCherry*.*nls* or *UAS-mCD8*::*GFP* was introduced into the 3rd chromosome when offspring's PGs were observed using histochemistry
** Offsprings carrying GFP-balancer chromosome was removed in all experiment.
(XLSX)

S8 Table The primer sets used for qPCR. (XLSX)

## References

[1] Edgar BA, Zielke N, Gutierrez C (2014) Endocycles: a recurrent evolutionary innovation for post-mitotic cell growth. Nat Rev Mol Cell Biol 15:197–210. 10.1038/nrm3756 24556841

[2] Orr-Weaver TL (2015) When bigger is better: the role of polyploidy in organogenesis. Trends Genet 31:307–315. 10.1016/j.tig.2015.03.011 25921783 PMC4537166

[3] Pandit SK, Westendorp B, de Bruin A (2013) Physiological significance of polyploidization in mammalian cells. Trends Cell Biol 23:556–566. 10.1016/j.tcb.2013.06.002 23849927

[4] Fox DT, Duronio RJ (2013) Endoreplication and polyploidy: insights into development and disease. Development 140:3–12. 10.1242/dev.080531 23222436 PMC3513989

[5] Zack TI, Schumacher SE, Carter SL, Cherniack AD, Saksena G, Tabak B et al (2013) Pan-cancer patterns of somatic copy number alteration. Nat Genet 45:1134–1140. 10.1038/ng.2760 24071852 PMC3966983

[6] Niu N, Zhang J, Zhang N, Mercado-Uribe I, Tao F, Han Z et al (2016) Linking genomic reorganization to tumor initiation via the giant cell cycle. Oncogenesis 5:e281 10.1038/oncsis.2016.75 27991913 PMC5177773

[7] Niu N, Mercado-Uribe I, Liu J (2017) Dedifferentiation into blastomere-like cancer stem cells via formation of polyploid giant cancer cells. Oncogene 36:4887–4900. 10.1038/onc.2017.72 28436947 PMC5582213

[8] Vitale I, Galluzzi L, Senovilla L, Criollo A, Jemaà M, Castedo M et al (2011) Illicit survival of cancer cells during polyploidization and depolyploidization. Cell Death Differ 18:1403–1413. 10.1038/cdd.2010.145 21072053 PMC3178421

[9] Pines J (2011) Cubism and the cell cycle: the many faces of the APC/C. Nat Rev Mol Cell Biol 12:427–438. 10.1038/nrm3132 21633387

[10] Shcherbata HR, Althauser C, Findley SD, Ruohola-Baker H (2004) The mitotic-to-endocycle switch in *Drosophila* follicle cells is executed by Notch-dependent regulation of G1/S, G2/M and M/G1 cell-cycle transitions. Development 131:3169–3181. 10.1242/dev.01172 15175253

[11] Schaeffer V, Althauser C, Shcherbata HR, Deng WM, Ruohola-Baker H. (2004) Notch-dependent Fizzy-related/Hec1/Cdh1 expression is required for the mitotic-to-endocycle transition in Drosophila follicle cells. Curr Biol. 14:630–636. 10.1016/j.cub.2004.03.040 15062106

[12] Cohen E, Allen SR, Sawyer JK, Fox DT. (2018) Fizzy-Related dictates a cell cycle switch during organ repair and tissue growth responses in the *Drosophila* hindgut. Elife. 17: e38327. 10.7554/eLife.38327 PMC6130973 30117808

[13] Shu Z, Deng WM. (2017) Differential Regulation of Cyclin E by Yorkie-Scalloped Signaling in Organ Development. G3 (Bethesda). 10:1049–1060. 10.1534/g3.117.039065 PMC5345706 28143945

[14] Djabrayan NJ, Cruz J, de Miguel C, Franch-Marro X, Casanova J. (2014) Specification of differentiated adult progenitors via inhibition of endocycle entry in the *Drosophila* trachea. Cell Rep. 9:859–865. 10.1016/j.celrep.2014.09.043 25437542

[15] Schoenfelder KP, Montague RA, Paramore SV, Lennox AL, Mahowald AP, Fox DT. (2014) Indispensable pre-mitotic endocycles promote aneuploidy in the *Drosophila* rectum. Development. 141:3551–3560. 10.1242/dev.109850 25142462 PMC6517832

[16] Imai KK, Ohashi Y, Tsuge T, Yoshizumi T, Matsui M, Oka A, Aoyama T. (2006) The A-type cyclin CYCA2;3 is a key regulator of ploidy levels in Arabidopsis endoreduplication. Plant Cell. 18:382–396. 10.1105/tpc.105.037309 16415207 PMC1356546

[17] Heyman J, Polyn S, Eekhout T, De Veylder L. (2017) Tissue-Specific Control of the Endocycle by the Anaphase Promoting Complex/Cyclosome Inhibitors UVI4 and DEL1. Plant Physiol. 175:303–313. 10.1104/pp.17.00785 28698355 PMC5580769

[18] Naoe H, Chiyoda T, Ishizawa J, Masuda K, Saya H, Kuninaka S. (2013) The APC/C activator Cdh1 regulates the G2/M transition during differentiation of placental trophoblast stem cells. Biochem Biophys Res Commun. 430:757–762. 10.1016/j.bbrc.2012.11.075 23206702

[19] Niwa YS, Niwa R (2016) Transcriptional regulation of insect steroid hormone biosynthesis and its role in controlling timing of molting and metamorphosis. Dev Growth Differ 58:94–105. 10.1111/dgd.12248 26667894 PMC11520982

[20] Yamanaka N, Rewitz KF, O'Connor MB (2013) Ecdysone control of developmental transitions: lessons from *Drosophila* research. Annu Rev Entomol 58:497–516. 10.1146/annurev-ento-120811-153608 23072462 PMC4060523

[21] Ou Q, Zeng J, Yamanaka N, Brakken-Thal C, O'Connor MB, King-Jones K (2016) The insect prothoracic gland as a model for steroid hormone biosynthesis and regulation. Cell Rep 16:247–262. 10.1016/j.celrep.2016.05.053 27320926 PMC5333552

[22] Ohhara Y, Kobayashi S, Yamanaka N (2017) Nutrient-dependent endocycling in steroidogenic tissue dictates timing of metamorphosis in *Drosophila melanogaster*. PLoS Genet 13:e1006583 10.1371/journal.pgen.1006583 28121986 PMC5298324

[23] Marchal E, Vandersmissen HP, Badisco L, Van de Velde S, Verlinden H, Iga M, et al (2010) Control of ecdysteroidogenesis in prothoracic glands of insects: a review. Peptides. 31:506–519. 10.1016/j.peptides.2009.08.020 19723550

[24] Zielke N, Korzelius J, van Straaten M, Bender K, Schuhknecht GF, Dutta D et al (2014) Fly-FUCCI: A versatile tool for studying cell proliferation in complex tissues. Cell Rep. 7:588–598. 10.1016/j.celrep.2014.03.020 24726363

[25] Casas-Tintó S, Arnés M, Ferrús A. (2017) *Drosophila* enhancer-Gal4 lines show ectopic expression during development. R Soc Open Sci. 4:170039 10.1098/rsos.170039 28405401 PMC5383858

[26] Danielsen ET, Moeller ME, Yamanaka N, Ou Q, Laursen JM, Soenderholm C et al (2016) A Drosophila genome-wide screen identifies regulators of steroid hormone production and developmental timing. Dev Cell 37:558–570. 10.1016/j.devcel.2016.05.015 27326933 PMC4918455

[27] Ohhara Y, Shimada-Niwa Y, Niwa R, Kayashima Y, Hayashi Y, Akagi K et al (2015) Autocrine regulation of ecdysone synthesis by β3-octopamine receptor in the prothoracic gland is essential for Drosophila metamorphosis. Proc Natl Acad Sci U S A 112:1452–1457. 10.1073/pnas.1414966112 25605909 PMC4321272

[28] Leroux MR, Hartl FU (2000) Protein folding: versatility of the cytosolic chaperonin TRiC/CCT. Curr Biol 10:R260–264. 10753735 10.1016/s0960-9822(00)00432-2

[29] Lundin VF, Leroux MR, Stirling PC (2010) Quality control of cytoskeletal proteins and human disease. Trends Biochem Sci 35:288–297 10.1016/j.tibs.2009.12.007 20116259

[30] Cong Y, Baker ML, Jakana J, Woolford D, Miller EJ, Reissmann S et al (2010) 4.0-Å resolution cryo-EM structure of the mammalian chaperonin TRiC/CCT reveals its unique subunit arrangement. Proc Natl Acad Sci U S A 107:4967–4972. 10.1073/pnas.0913774107 20194787 PMC2841888

[31] Kaisari S, Sitry-Shevah D, Miniowitz-Shemtov S, Teichner A, Hershko A. (2017) Role of CCT chaperonin in the disassembly of mitotic checkpoint complexes. Proc Natl Acad Sci U S A. 114:956–961. 10.1073/pnas.1620451114 28096334 PMC5293070

[32] Camasses A, Bogdanova A, Shevchenko A, Zachariae W (2003) The CCT chaperonin promotes activation of the anaphase-promoting complex through the generation of functional Cdc20. Mol Cell 12:87–100. 12887895 10.1016/s1097-2765(03)00244-2

[33] Zielke N, Querings S, Rottig C, Lehner C, Sprenger F (2008) The anaphase-promoting complex/cyclosome (APC/C) is required for rereplication control in endoreplication cycles. Genes Dev 22:1690–1703. 10.1101/gad.469108 18559483 PMC2428065

[34] Narbonne-Reveau K, Senger S, Pal M, Herr A, Richardson HE, Asano M et al (2008) APC/CFzr/Cdh1 promotes cell cycle progression during the Drosophila endocycle. Development 135:1451–1461. 10.1242/dev.016295 18321983

[35] Sallé J, Campbell SD, Gho M, Audibert A (2012) CycA is involved in the control of endoreplication dynamics in the *Drosophila* bristle lineage. Development 139:547–557. 10.1242/dev.069823 22223681

[36] Sánchez-Higueras C, Sotillos S, Castelli-Gair Hombría J (2014) Common origin of insect trachea and endocrine organs from a segmentally repeated precursor. Curr Biol 24:76–81. 10.1016/j.cub.2013.11.010 24332544

[37] Roh SH, Kasembeli M, Bakthavatsalam D, Chiu W, Tweardy DJ (2015) Contribution of the Type II Chaperonin, TRiC/CCT, to Oncogenesis. Int J Mol Sci 16:26706–26720. 10.3390/ijms161125975 26561808 PMC4661834

[38] Schindelin J, Arganda-Carreras I, Frise E, Kaynig V, Longair M, Pietzsch T et al (2012) Fiji: an open-source platform for biological-image analysis. Nat Methods 9:676–682. 10.1038/nmeth.2019 22743772 PMC3855844

[39] Losick VP, Fox DT, Spradling AC. (2013) Polyploidization and cell fusion contribute to wound healing in the adult *Drosophila* epithelium. Curr Biol. 23:2224–2232. 10.1016/j.cub.2013.09.029 24184101 PMC3898104

