# Supplementary figures and images for: Chaperonin TRiC/CCT supports mitotic exit and entry into endocycle in Drosophila
Source: PLoS Genet. 2019 Apr 29;15(4):e1008121. doi: 10.1371/journal.pgen.1008121 (PMC6508744; doi:10.1371/journal.pgen.1008121)

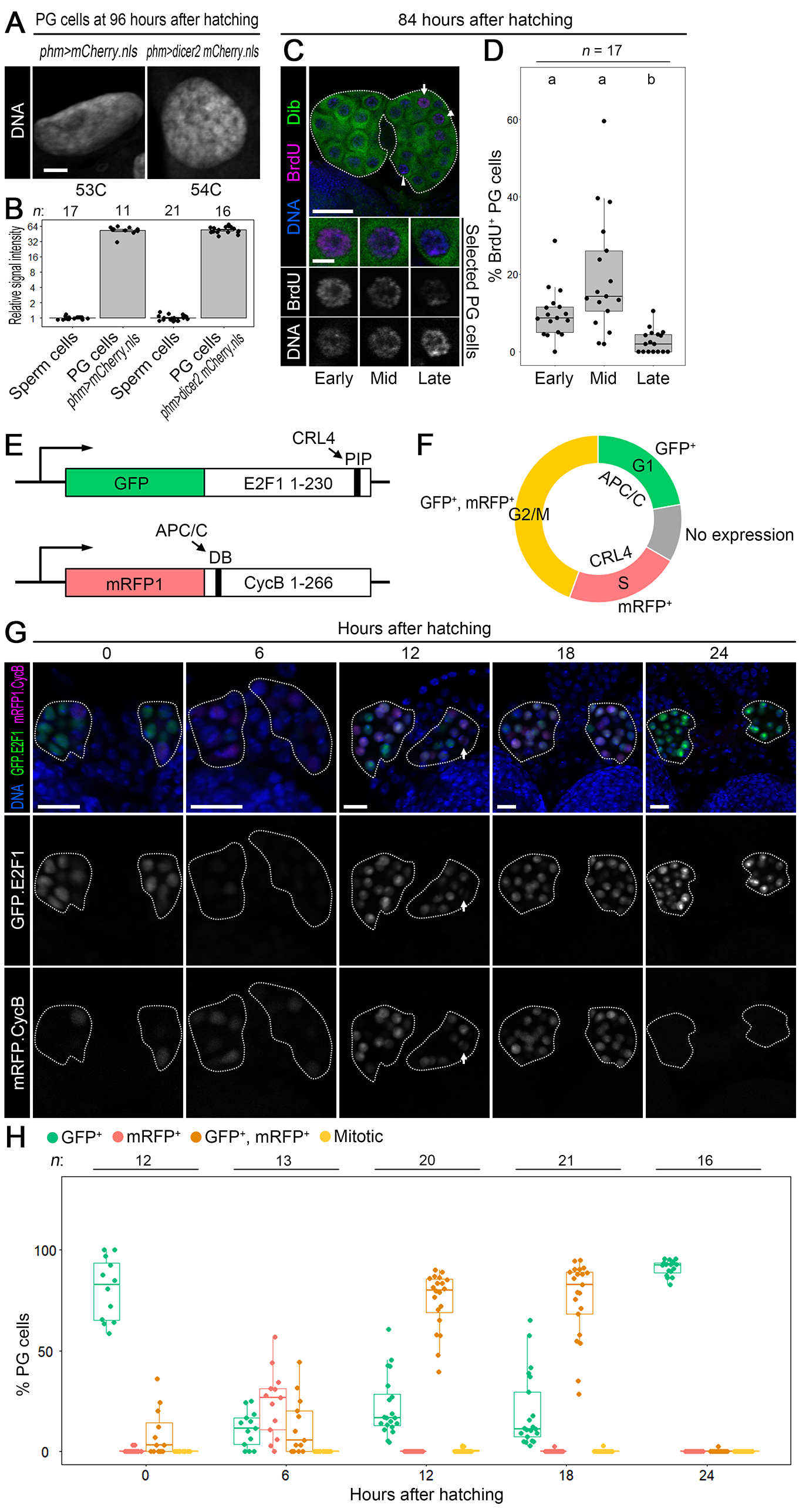

Supplement: S1 Fig — (A) Sperm cells of Oregon R (left panel) and PG cells at 96 hAH of control [phm>mCherry.nls (middle panel) and phm>dicer2 mCherry.nls (right panel)]. DNA was stained by Hoechst. Scale bars: 10 μm. (B) Scatter plots with mean value of relative signal intensity of Hoechst in Oregon R sperm cells and PG cells of control. The mean C value in sperm cells was normalized to 1, and accordingly, the mean values in PG cells of phm>mCherry.nls and phm>dicer2 mCherry.nls at 96 hAH were set to 53 and 54, respectively. Sample sizes (the number of sperm and PG cells) are shown above each column. (C) Incorporation of BrdU in the PG at 84 hAH. Incorporated BrdU was detected by anti-BrdU antibody (magenta in upper panel), the PG cell was stained using specific antibody against Dib (green in upper panel), and DNA was detected by Hoechst (blue in upper panel). The PG is indicated by dotted line. Early, middle (mid), and late-S phase cells were indicated by the arrow, arrowhead, and sharp arrowhead, respectively, and the zoomed images of these cells were shown in the lower panels. Scale bars: 50 μm (upper panel) and 10 μm (lower panels). (D) Scatter and box plots showing the percentage of early, mid, and late-S phase PG cells at 96 hAH. Different lowercase letters indicate statistically significant differences (P < 0.05; Steel–Dwass test, see S2 Table). Box and dot plots as in Fig 1C–1E. Sample sizes (the number of PGs) are shown above column. (E and F) Schematics of Drosophila FUCCI system. E2F11-230-fused GFP (GFP.E2F1) and CycB1-266-fused mRFP1 (mRFP1.CycB) expressed under the control of Gal4/UAS system were degraded through CRL4- and APC/C-dependent manner, respectively (E). Since CRL4 and APC/C-dependent protein degradation are active at S and G1 phase in mitotic cell cycle, G1-, S-, and G2/M-phase cells were labelled by GFP, mRFP1, and both GFP and mRFP1, respectively (F). (G) The expression patterns of GFP.E2F1 (green and white in the upper and middle panels, respectively) and mR [file pgen.1008121.s002.tif]

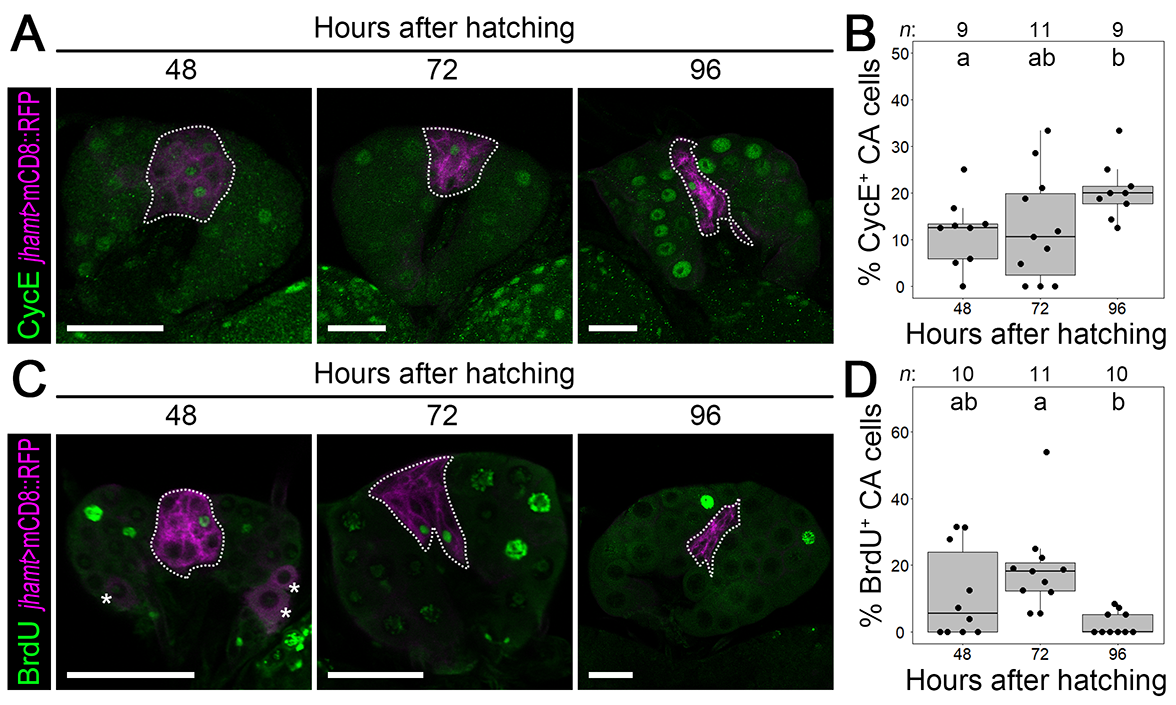

Supplement: S2 Fig — (A) CycE expression in the CA. CycE was detected by anti-CycE antibody (green), and the CA was labeled by mCD8-fused RFP (mCD8::RFP; magenta) expressed under the control of jhamt-Gal4, at indicated time points. The CAs are indicated by dotted lines. Scale bars: 50 μm. (B) Scatter and box plots showing the percentage of CycE-positive CA cells at indicated stages. Different lowercase letters indicate statistically significant differences (P < 0.05; Steel–Dwass test; see S2 Table). Sample sizes (the number of CAs) are shown above each column. (C) Incorporation of BrdU in the CA. Incorporated BrdU was detected by anti-BrdU antibody (green), and the CA was labeled by mRFP (magenta) at indicated time points. The CAs are indicated by dotted lines. Scale bars: 50 μm. (D) Scatter and box plots showing the percentage of BrdU-positive CA cells at indicated stages. Different lowercase letters indicate statistically significant differences (P < 0.05; Steel–Dwass test; see S2 Table). Sample sizes (the number of CAs) are shown above each column. (TIF) [file pgen.1008121.s003.tif]

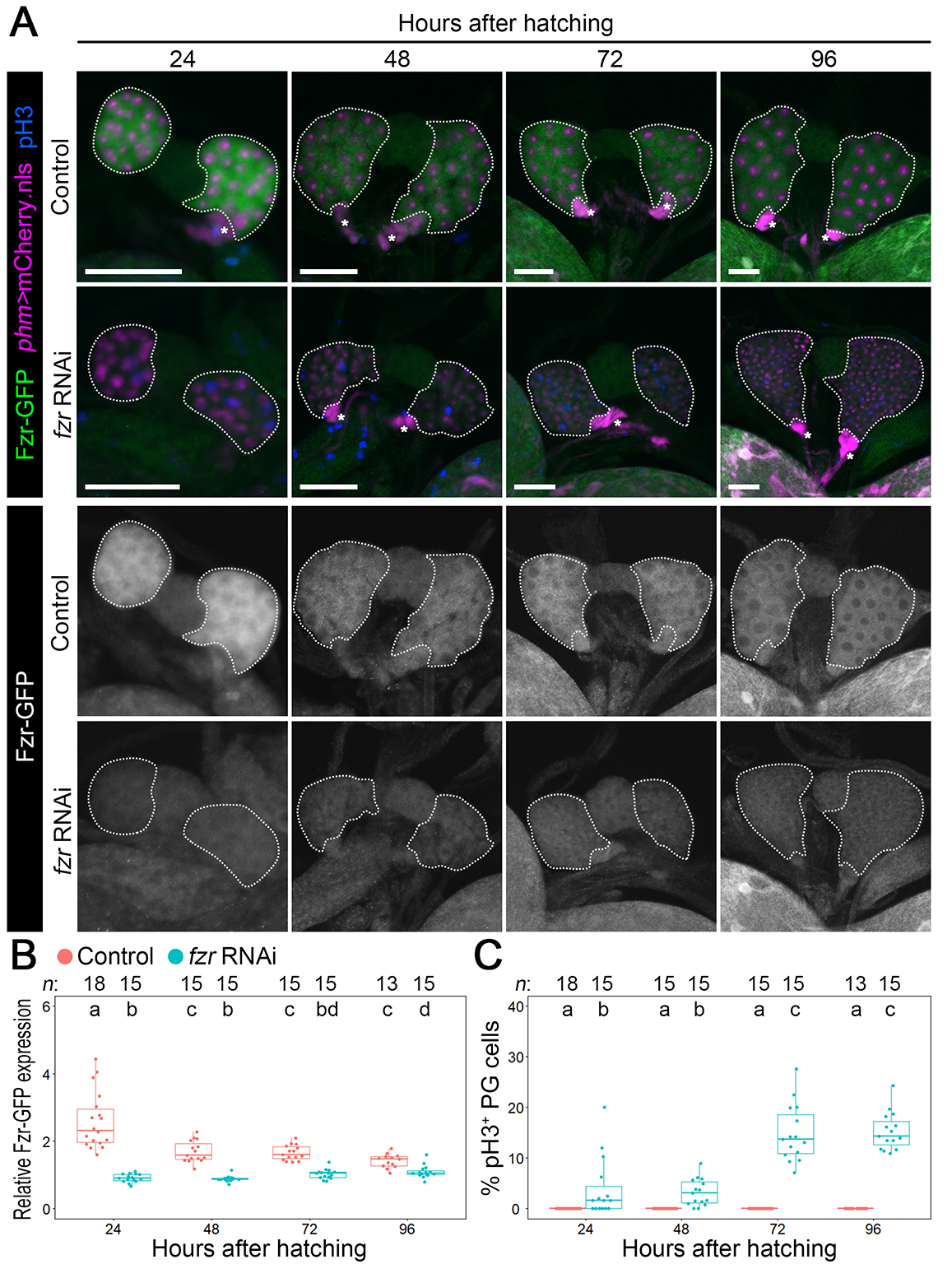

Supplement: S3 Fig — (A) Fzr-GFP expression (green and white in the upper and lower panels, respectively) in the PG of the controls (phm>dicer2 mCherry.nls, fzr-GFP) and fzr RNAi animals (phm>dicer2 mCherry.nls fzrRNAi, fzr-GFP) at indicated time points. PG cells were labeled by mCherry.nls (magenta in the upper panels), and pH3 was stained by specific antibody to pH3 (blue in the upper panels). The PGs are indicated by dotted lines. The asterisks indicate CC cells expressing 3xP3-dsRed marker in Fzr-GFP transgene. Scale bars: 50 μm. (B and C) Scatter and box plots showing the relative expression of Fzr-GFP (B) and the percentage of pH3-positive cells (C) in the PG of the controls (red) and fzr RNAi (blue) at indicated stages. Different lowercase letters indicate statistically significant differences (P < 0.05; Steel–Dwass test; see S2 Table). Sample sizes (the number of PGs) are shown above each column. (TIF) [file pgen.1008121.s004.tif]

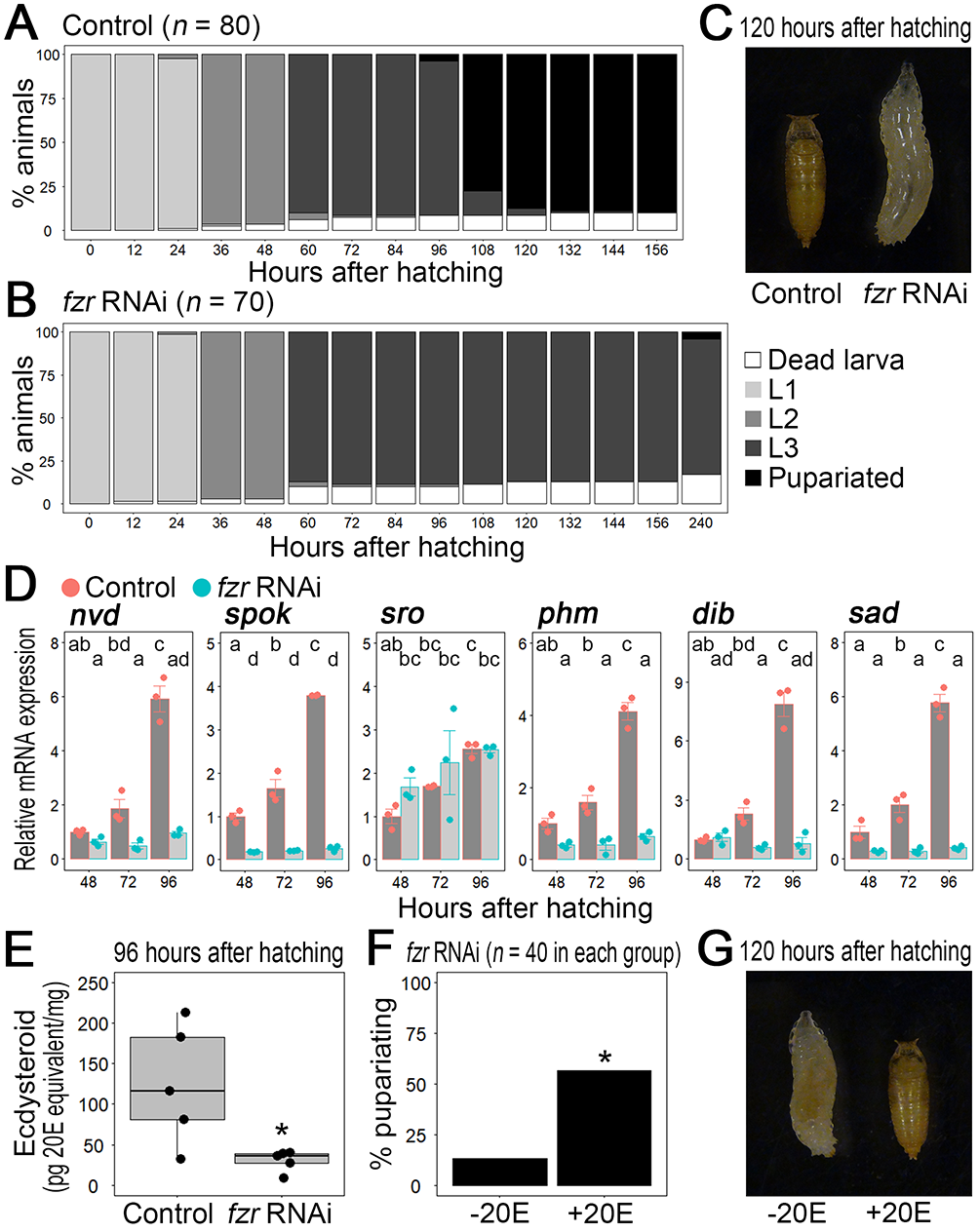

Supplement: S4 Fig — (A and B) Percentages of L1, L2, L3, and pupariated animals in the controls (phm>dicer2) and fzr RNAi (phm>dicer2 fzrRNAi) during development. Sample sizes (the number of animals) are indicated in parentheses. (C) Pupariated control and fzr RNAi arrested at the L3 stage. (D) The expression level of ecdysone biosynthetic genes in the controls and fzr RNAi measured using qPCR at indicated time points. Average values of triplicate data sets with SE and scatter plots are shown. Ten to fifteen larvae were pooled in each datum. Different lowercase letters indicate statistically significant differences (P < 0.05; Tukey’s multiple comparison test; see S2 Table). (E) Whole-body ecdysteroid levels in the controls and fzr RNAi animals at 96 hAH measured using ELISA. Ecdysteroid levels of five independent data sets are shown by scatter and box plots. Ten larvae were pooled in each datum. The asterisk indicates statistically significant differences (P < 0.05; Welch’s two sample t-test). (F) Percentages of pupariated fzr RNAi animals cultured on the medium with 20E (0.5 mg/g) or without 20E from 48 hAH. Sample sizes (the number of animals) are indicated in parentheses. The asterisk indicates statistically significant differences (P < 0.05; Fisher’s test). (G) fzr RNAi larva fed on -20E medium and pupariated fzr RNAi animal fed on +20E medium. (TIF) [file pgen.1008121.s005.tif]

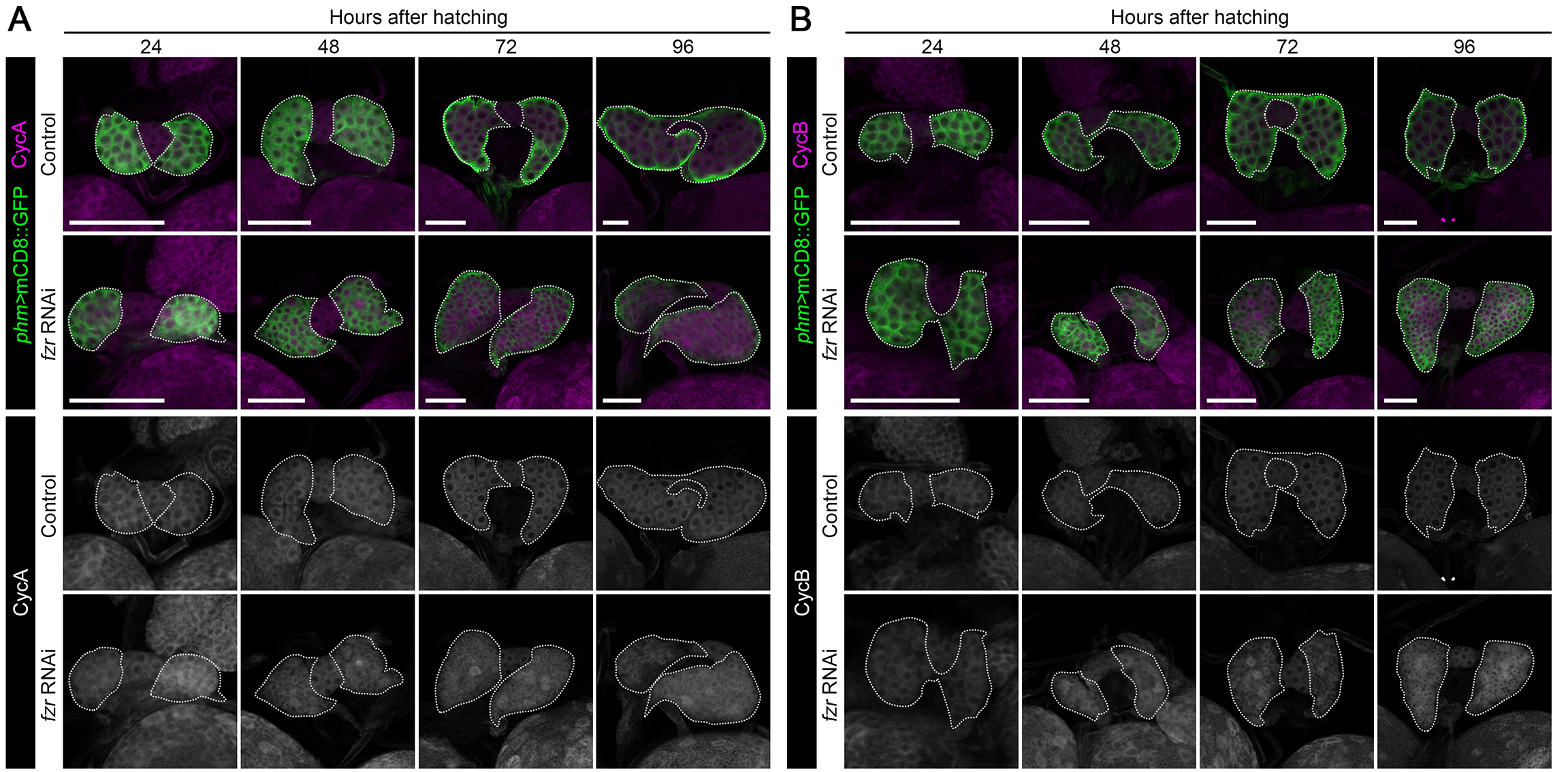

Supplement: S5 Fig — CycA (A) and B expression (B) in the PG of the controls (phm>dicer2 mCD8::GFP) and fzr RNAi larvae (phm>dicer2 mCD8::GFP fzr-RNAi) at indicated time points. PG cells were labelled by mCD8::GFP (green in the upper panels) and CycA and B were detected by specific antibodies against each cyclin (magenta and white in the upper and lower panels, respectively). The PGs are indicated by dotted lines. Scale bars: 50 μm. The percentage of CycA and B-positive PG cells of control and fzr RNAi at 24, 48, 72, and 96 hAH is summarized in Fig 3C and 3D. (TIF) [file pgen.1008121.s006.tif]

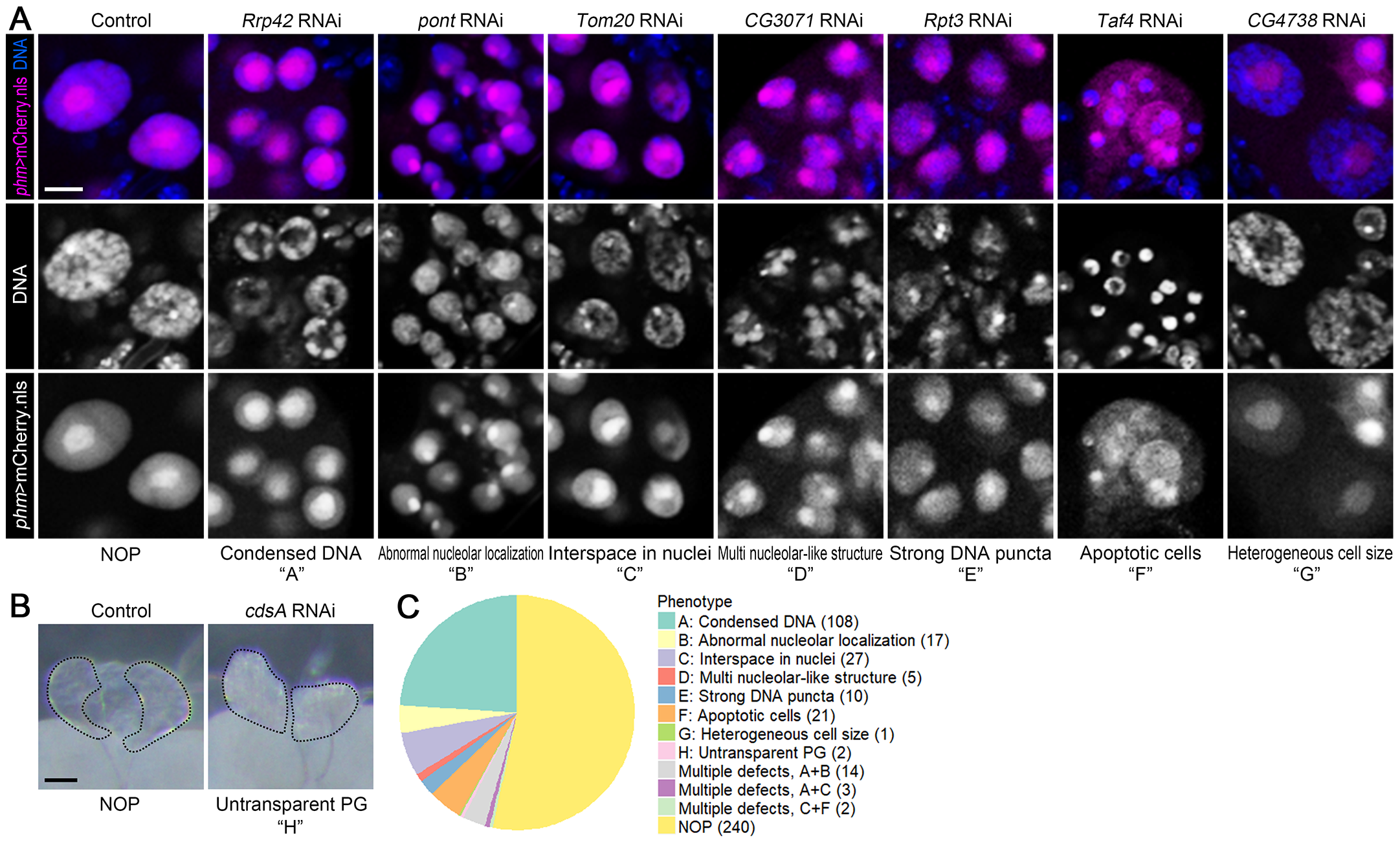

Supplement: S6 Fig — (A) PG cells of the controls (phm>mCherry.nls) and RNAi animals (phm>mCherry.nls gene-of-interest-RNAi) showing morphological defects. Each phenotypic groups are categorized into “A”–”G” groups as indicated in lower comments. DNA was stained by Hoechst (blue and white in the upper and middle panels, respectively), and the nuclei of PG cells were labelled by mCherry.nls (magenta and white in the upper and lower panels, respectively). The arrows, arrowheads, and narrow arrowheads indicate interspace in the nuclei, duplicated nucleolar-like structure, and strong DNA puncta, respectively. Scale bar: 10 μm. (B) The PG of control and cdsA RNAi. The PG of cdsA RNAi is untransparent compared to control, which is categorized as “H” in this screening. The PGs are indicated by dotted lines. Scale bar: 50 μm. (C) Pie chart showing the distribution of the phenotypic categories of morphological defects in PG cells. Sample sizes (the number of animals) are indicated in parentheses. (TIF) [file pgen.1008121.s007.tif]

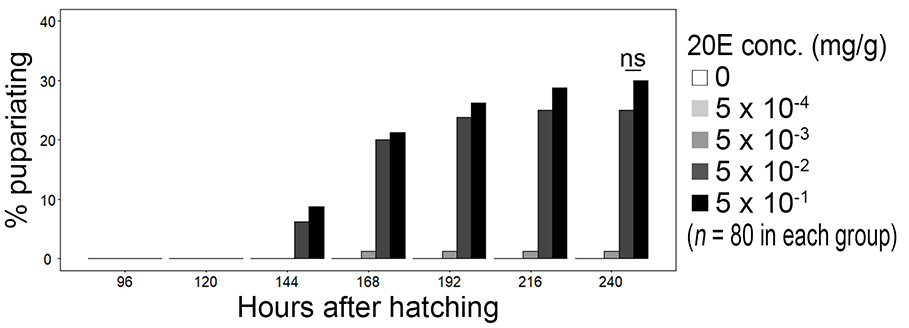

Supplement: S7 Fig — The percentages of pupariated cct8 RNAi animals, cultured on the medium with 20E (5 x 10^−4^, 5 x 10^−3^, 5 x 10^−2^, and 5 x 10−1 mg/g) or without 20E from 48 hAH, at indicated stages. Sample sizes (the number of animals) are indicated in parentheses. ns, not significant (Fisher’s test, P > 0.05). (TIF) [file pgen.1008121.s008.tif]

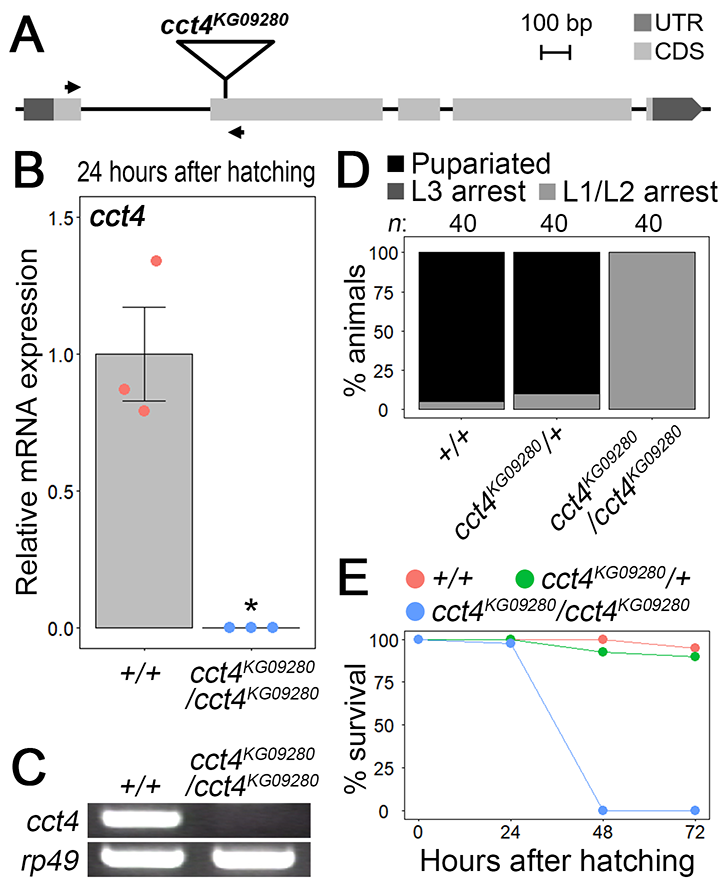

Supplement: S8 Fig — (A) Schematic diagram of cct4 gene region and KG09280 insertion site. The arrows indicate the primer sets used for qPCR to measure cct4 expression level. (B) The relative expression level of cct4 in wild-type (+/+) and cct4KG09280 homozygous mutant (cct4KG09280/cct4KG09280) measured using qPCR at 24 hAH. Average values of triplicate data sets with SE and scatter plots are shown. The asterisk indicates statistically significant differences (P < 0.05; Welch’s two sample t-test). (C) Agarose gel electrophoresis of PCR product of rp49 and cct4 in wild-type and cct4KG09280. (D) Percentages of pupariated and L1/L2- and L3-arrested animals in wild-type and cct4KG09280 heterozygous and homozygous mutant are shown. Sample size (the number of animals) are shown above each column. (E) Percentages of survival in wild-type and cct4KG09280 heterozygous and homozygous mutant are shown at indicated time points. Sample sizes are the same as in D. (TIF) [file pgen.1008121.s009.tif]

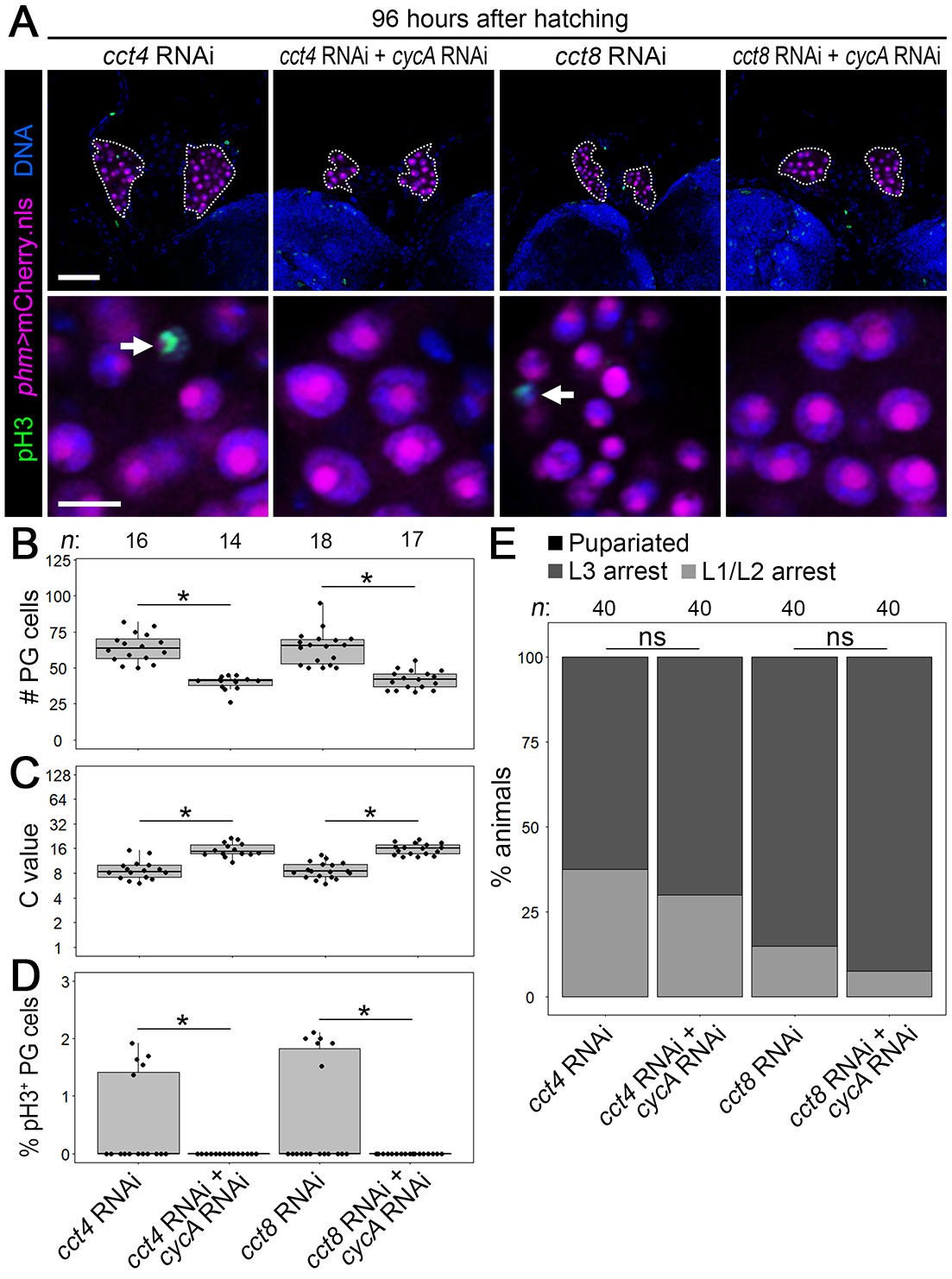

Supplement: S9 Fig — (A) The PG (upper panels) and PG cells in higher magnification (lower panels) of cct4 RNAi (phm>mCherry.nls cct4-RNAi), cct4 RNAi + cycA RNAi (phm>mCherry.nls cct4-RNAi cycA-RNAi), cct8 RNAi (phm>mCherry.nls cct8-RNAi), and cct8 RNAi + cycA RNAi (phm>mCherry.nls cct8-RNAi cycA-RNAi) at 96 hAH. pH3 and DNA were detected by anti-pH3 antibody (green) and Hoechst (blue), respectively, and the nuclei of PG cells were labelled by mCherry.nls (magenta). The PGs are indicated by dotted lines. The arrows indicate pH3-positive PG cells. Scale bars: 50 μm (upper panels) and 10 μm (lower panels). (B–D) Scatter and box plots showing the cell number (B), the C value (C), and the percentage of pH3-positive cells (D) in the PG of cct4 RNAi, cct4 RNAi + cycA RNAi, cct8 RNAi, and cct8 RNAi + cycA RNAi animals at 96 hAH. The asterisks indicate statistically significant differences (P < 0.05; Mann–Whitney U test). Box and dot plots as in Fig 1C–1E. The mean C values of cct4 and cct8 RNAi at 96 hAH were set to 9 and 8.8, according to their mean C value at the same time point (Fig 6C). Sample size (the number of PGs) are shown above each column. (E) Percentages of pupariated and L1/L2- and L3-arrested animals in cct4 RNAi, cct4 RNAi + cycA RNAi, cct8 RNAi, and cct8 RNAi + cycA RNAi are shown. Sample sizes (the number of animals) are shown above each column. ns, not significant (P > 0.05; Fisher’s test). (TIF) [file pgen.1008121.s010.tif]
